# Metabolic Milieu and Susceptibility: The Combined Benefits of Targeting AMPK and ACE2 for Metabolic Disorders and COVID-19

**DOI:** 10.3390/ijms27208997

**Published:** 2026-10-11

**Authors:** Fave Yohanna Tata, Mthokozisi Bongani Nxumalo, Nosipho Ntanzi, Aganze Gloire-Aimé Mushebenge, René Bernadette Khan, Hezekiel Mathambo Kumalo

**Affiliations:** 1 Drug Research and Innovation Unit, Discipline of Medical Biochemistry, School of Laboratory Medicine and Medical Science, University of KwaZulu-Natal, Durban 4000, South Africakhanr@ukzn.ac.za (R.B.K.); kumaloh@ukzn.ac.za (H.M.K.); 2 Molecular and Clinical Pharmacology Research Laboratory, Department of Pharmacology, Discipline of Pharmaceutical Sciences, University of KwaZulu-Natal, P.O. Box X5401, Durban 4000, South Africa; 3 Department of Health Studies, College of Human Sciences, Muckleneuk Campus, University of South Africa, Pretoria 0027, South Africa; musheag@unisa.ac.za

**Keywords:** cardiometabolic diseases, flavonoids, viral infection, COVID-19, ACE2, AMPK, diabetes

## Abstract

The convergence of metabolic dysfunction and infectious diseases has become increasingly evident, particularly during the COVID-19 pandemic. AMP-activated protein kinase (AMPK) and angiotensin-converting enzyme 2 (ACE2) emerge as pivotal molecular regulators linking metabolic homeostasis, endothelial integrity, immune modulation, and viral pathogenesis. Metabolic diseases such as diabetes, obesity, and non-alcoholic fatty liver disease are marked by insulin resistance, chronic inflammation, oxidative stress, and endothelial dysfunction, collectively increasing susceptibility to severe viral infections and outcomes. This review examines experimental, computational, and translational evidence exploring the interplay between AMPK and ACE2, focusing on metabolic, vascular, and inflammatory pathways relevant to viral infections. Recent findings show a bidirectional functional relationship between AMPK and ACE2, where AMPK phosphorylation supports ACE2 expression and function, and ACE2 signaling reciprocally enhances AMPK activity, forming a protective metabolic–vascular loop. Disruption of this axis, particularly through SARS-CoV-2-induced ACE2 degradation, exacerbates metabolic deterioration, vascular damage, and exaggerated inflammatory responses. Preclinical and in silico studies show that pharmacological AMPK activators, including metformin and bioactive flavonoids, demonstrate pleiotropic metabolic, anti-inflammatory, endothelial protective, and potential antiviral activities, hinting at the therapeutic potential of modulating the AMPK-ACE2 axis, despite the limited clinical application of flavonoids. The AMPK-ACE2 axis represents an important molecular link between metabolic regulation, renovascular health, and immune defense mechanisms. This evaluation connects AMPK and ACE2 from isolated signaling nodes into a coordinated pharmacological axis with implications for interventions in cardiometabolic and viral diseases. Future research should prioritize mechanistic validation and conduct integrated experimental and clinical investigations to establish AMPK-ACE2 co-targeting as a viable therapeutic strategy for mitigating cardiometabolic disease burden and enhancing resilience against viral infections.

## 1. Introduction

Metabolic diseases are chronic disorders arising from disruptions in biochemical and enzymatic pathways, leading to systemic dysregulation and impaired energy metabolism, accumulation of toxic metabolites, and multi-organ dysfunction. These chronic disorders encompass a wide range of conditions, including diabetes mellitus (DM), obesity, non-alcoholic fatty liver disease (NAFLD), chronic kidney disease (CKD), and lipid metabolism disorders, all of which are significant global health burdens, contributing to high morbidity, mortality, and disability-adjusted life years [1,2]. Central mechanisms underlying these disorders are chronic inflammation, insulin resistance, and oxidative stress, often accompanied by endothelial impairment, which collectively drive systemic metabolic dysregulation [3,4]. The increasing prevalence of these conditions is largely attributable to unhealthy dietary habits, physical inactivity, genetic susceptibility, and population aging [5,6]. These risk factors contribute to insulin resistance and visceral adiposity, which are central to the development of T2DM and metabolic syndrome [7,8]. Converging evidence also implicates gut dysbiosis and disruption of the intestinal barrier in contributing to insulin resistance and systemic inflammation [9,10]. Similarly, epigenetic mechanisms further influence metabolic susceptibility by affecting adipose distribution and insulin sensitivity [11,12].

The COVID-19 pandemic exposed an alarming intersection between infection and metabolism, as individuals with pre-existing metabolic diseases such as diabetes, obesity, or hypertension experienced disproportionately severe disease outcomes, including higher hospitalization and mortality rates [13,14,15]. Mechanistic links point to viral cellular entry through the angiotensin-converting enzyme 2 (ACE2) receptor and disturbances in key metabolic regulators, such as AMP-activated protein kinase (AMPK), which ultimately compromise immune responses, vascular health, and energy homeostasis. Both ACE2 and AMPK are critical regulatory nodes with essential roles in metabolic regulation, cardiovascular health, and disease pathogenesis, including COVID-19 [16]. AMPK is a central energy sensor that maintains cellular energy homeostasis, whereas ACE2 serves as a counter-regulatory enzyme within the renin–angiotensin system (RAS), protecting against cardiovascular and metabolic injury. Their synergistic roles in energy balance, endothelial integrity, and immune modulation underscore their therapeutic relevance. Importantly, the interplay between AMPK and ACE2 presents a compelling pharmacological target not only for mitigating cardiometabolic disorders but also for reducing the severity of viral infections, such as COVID-19. This therapeutic modulation of the AMPK-ACE2 axis may offer dual benefits. Hence, a deeper understanding of this regulatory axis could accelerate the development of novel therapeutics for cardiometabolic disorders while minimizing toxicity and improving resilience to infectious diseases.

Computational approaches are increasingly recognized as effective tools for accelerating the discovery of therapeutics targeting complex molecular networks. Computational techniques like molecular docking, molecular dynamics (MD) simulations, and binding free energy calculations (MM/GBSA or MM/PBSA) are helpful in identifying and optimizing compounds that interact with proteins regulating multiple signaling pathways [17,18], including the AMPK and ACE2 regulatory axis. These methods are particularly useful for screening phytochemicals and small molecules that stabilize protein–ligand interactions and modulate regulatory domains to influence protein functions for therapeutic benefits. By employing computational methods, compounds can be identified that interact with AMPK regulatory sites while also influencing ACE2 activity and stability, thereby connecting AMPK signaling and ACE2 activity. Integrating computational screening with experimental investigations is a promising strategy for identifying multi-target modulators that could enhance the protective metabolic and vascular pathways through the AMPK-ACE2 axis.

## 2. Metabolic Dysregulation and Pathophysiological Nexus in Metabolic Diseases

**Figure 1:**
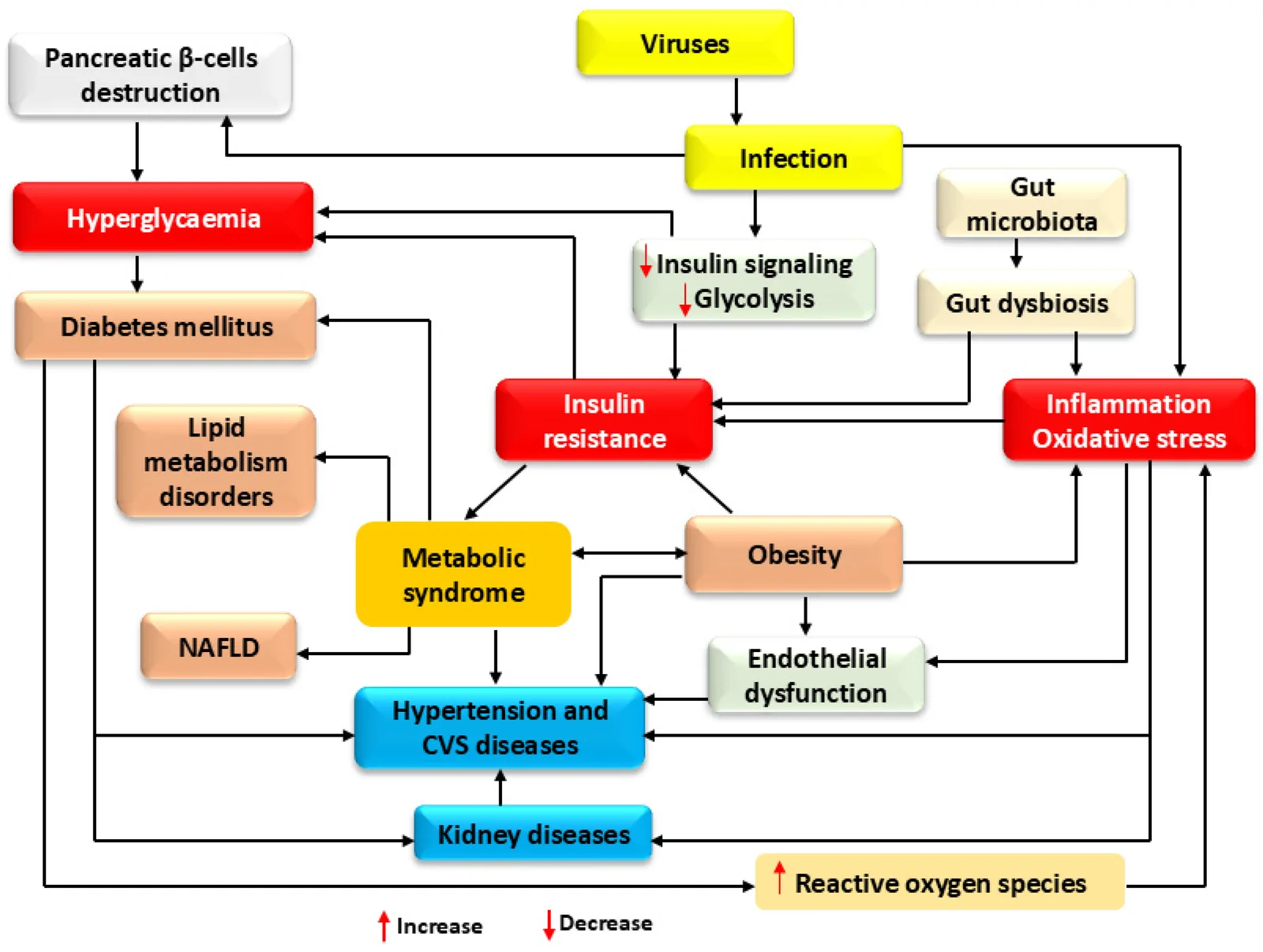
Pathogenic and pathophysiological nexus in metabolic diseases.

Metabolic diseases arise from a complex interplay of chronic inflammation, oxidative stress, endothelial dysfunction, and metabolic disruptions. These factors are central to the pathogenesis and progression of conditions such as obesity, type 2 diabetes, and metabolic syndrome, which can lead to cardiovascular morbidity and complications [3]. Key metabolic abnormalities to these disorders are insulin resistance, hyperglycemia, and dyslipidemia, intricately linked to inflammation and endothelial dysfunction [19,20]. Diabetes, characterized by persistent hyperglycemia resulting from loss of insulin secretion, insulin resistance, and disrupted glucose metabolism, is primarily influenced by genetic and lifestyle factors [21,22]. However, viral infections also play significant roles in the onset and progression of diabetes (Figure 1). Viruses can infect pancreatic β-cells, trigger β-cell autoimmunity through molecular mimicry, and disrupt insulin signaling. In type 1 diabetes mellitus (T1DM), viruses can lead to β-cell dysfunction or destruction through autoimmunity involving T-cell cross-reactivity with viral antigens and glutamic acid decarboxylase 65 (GAD65). In type 2 diabetes mellitus (T2DM), viral infections worsen metabolic dysfunction, inflammation, insulin resistance, and oxidative stress. Viruses disrupt insulin signaling pathways by inhibiting insulin receptor substrate (IRS) phosphorylation, suppression of FOXO-mediated gene transcription, and impaired translocation of GLUT4, reducing glucose uptake and promoting disease progression [23,24]. The relationship between viral infections and diabetes is complex and bidirectional. Viral infections induce chronic low-grade inflammation, oxidative stress, and gut microbiome dysbiosis, while hyperglycemia in diabetes enhances viral pathogenicity by glycosylating viral spike proteins, increasing their binding affinity for the ACE2 receptor and facilitating viral entry. Moreover, hyperglycemia impairs immune responses, diminishing phagocytosis and increasing the release of pro-inflammatory cytokines, exacerbating systemic inflammation [24,25]. Inflammation plays a central role in disrupting insulin signaling and metabolic processes. Pro-inflammatory cytokines, such as tumor necrosis factor-α (TNF-α), interleukin-6 (IL-6), and interleukin-1β (IL-1β), can induce insulin resistance by promoting serine phosphorylation of insulin receptor substrate (IRS) proteins. Impaired insulin signaling also activates inflammatory signaling pathways, such as nuclear factor-kappa B (NF-κB) and c-Jun N-terminal kinase (JNK). These cascades contribute to β-cell dysfunction and oxidative damage, culminating in endothelial injury and metabolic dysregulation in T2DM [26,27]. Type 2 diabetes, characterized by chronic hyperglycemia and insulin resistance, increases the risk of severe cardiovascular, renal, and neurological complications [28,29]. Furthermore, obesity, characterized by excessive adiposity and a major risk factor, is strongly associated with systemic inflammation, insulin resistance, and dyslipidemia, which are key components of metabolic syndrome [30]. Metabolic syndrome comprises a cluster of interrelated cardiometabolic risk factors, like central obesity, insulin resistance, dyslipidemia, and hypertension, which synergistically increase the risk of T2DM, cardiovascular diseases, and non-alcoholic liver disease (NAFLD) [20,29]. Its pathogenesis is multifactorial, involving autonomic dysfunction, chronic inflammation, and changes in gut microbiota. Gut microbiota dysbiosis can lead to the production of harmful microbial metabolites, such as lipopolysaccharides, that promote inflammation and insulin resistance (Figure 1), contributing to diabetes [31].

## 3. Insulin Resistance and Immunovascular Disruptions

Insulin resistance is a key pathological factor at the core of metabolic disorders such as T2DM, obesity, metabolic syndrome, and cardiovascular diseases. Insulin resistance caused by disruption of insulin signaling in skeletal muscle, liver, and adipose tissue contributes to hyperglycemia and dyslipidemia. It impairs nitric oxide (NO) production through PI3K/Akt/eNOS pathways, leading to vasoconstriction, vascular inflammation, atherosclerosis, and the development of cardiovascular complications associated with diabetes. Dyslipidemia promotes endothelial function through enhanced reactive oxygen species (ROS) production and decreased nitric oxide (NO) bioavailability [32], while chronic hyperglycemia exacerbates metabolic dysfunction through multiple mechanisms, including glucose auto-oxidation, activation of hexosamine and protein kinase C (PKC) pathways, formation of advanced glycation products (AGEs), and increased angiotensin II activity [24,33]. Moreover, hyperglycemia impairs immune responses [34] and, along with hyperlipidemia and insulin resistance, enhances ROS generation. Elevated ROS degrades NO, impairs vasodilation, increases vascular permeability, and perpetuates endothelial dysfunction [35,36]. These processes synergistically induced oxidative stress, endothelial dysfunction, and inflammatory activation [35], increasing the risks of cardiovascular complications.

## 4. Adipose Tissue Dysfunction and Systemic Inflammation

Abnormal secretion of adipokines, such as low adiponectin levels and elevated leptin levels from dysfunctional adipose tissue, disrupts insulin signaling and promotes insulin resistance, metabolic disturbances, and inflammation. Chronic low-grade inflammation is a hallmark of metabolic diseases [37] and is largely driven by dysfunctional adipose tissue, which secretes pro-inflammatory adipokines and cytokines [4]. In obesity, adipose tissue hypoxia promotes macrophage infiltration and sustained cytokine release, which further exacerbates inflammation and tissue damage [32,38]. The activation of inflammasomes and the release of pro-inflammatory cytokines, such as TNF-α and IL-6, disrupt insulin signaling and promote visceral fat deposition, further perpetuating metabolic dysfunction [12,28]. Chronic inflammation and oxidative stress impair insulin receptor substrate-1 (IRS-1) signaling and promote insulin resistance and hepatic gluconeogenesis [39,40], which contribute to broader systemic inflammation and metabolic dysfunction. These inflammatory mediators also contribute to endothelial dysfunction and heighten cardiovascular risks.

## 5. Vascular Consequences of Endothelial Dysfunction and Metabolic Inflammation

The vascular endothelium, which is central to vascular tone regulation, permeability, and immune responses, is profoundly affected in metabolic diseases [41,42]. Endothelial dysfunction, characterized by decreased nitric oxide (NO) bioavailability and elevated ROS production, is a key mediator of cardiovascular pathologies, including atherosclerosis, hypertension, and cardiac dysfunction [32,43,44]. Hyperglycemia, hyperlipidemia, and insulin resistance contribute to this dysfunction by promoting oxidative stress and impairing vasodilation [35,36]. Moreover, endothelial dysfunction aggravates inflammation by promoting leukocyte recruitment and pro-inflammatory cytokine release [32,45]. Tumor necrosis factor alpha (TNF-α) and interleukin-6 (IL-6) upregulate adhesion molecules ICAM-1 and VCAM-1 on endothelial cells, facilitating immune cell infiltration and exacerbating vascular inflammation [40,46]. Insulin resistance also inhibits endothelial nitric oxide synthase (eNOS) activity, further reducing NO and impairing endothelial function [47,48]. These mechanisms may contribute to the heightened susceptibility of individuals with metabolic diseases to severe COVID-19 outcomes by creating a microenvironment favorable for viral replication and infectivity [41].

## 6. The Role of Gut Dysbiosis and Inflammation in Metabolic Dysregulation

Gut microbiota dysbiosis plays a vital role in the onset and progression of metabolic diseases. Reduced gut microbiota has been associated with low-grade inflammation, increased adiposity, insulin resistance, and altered energy metabolism, contributing to the development of obesity and T2DM [49]. Dysbiosis facilitates the production of pro-inflammatory intermediates and ROS, which exacerbate oxidative stress, insulin resistance, and impair metabolic homeostasis [12]. Thus, gut dysbiosis and disruption of the intestinal barrier contribute to systemic inflammation, insulin resistance, and broader metabolic dysregulation damage [9]. The interplay and interdependence of inflammation, endothelial dysfunction, and metabolic derangements create a vicious cycle that perpetuates disease progression.

## 7. Glucose Metabolism and Homeostasis

Glucose is an important metabolic substrate that serves as the primary source of ATP, the essential energy currency for normal cellular and organ function. It is derived from dietary carbohydrates and processes, such as hepatic glycogenolysis and gluconeogenesis, from amino acids and lactate. Once absorbed, glucose is metabolized through glycolysis to generate pyruvate, which is further oxidatively decarboxylated to form acetyl-CoA by pyruvate dehydrogenase in aerobic conditions. Acetyl-CoA then enters the tricarboxylic acid (TCA) cycle, ultimately generating CO_2_, H_2_O, and ATP, essential for cellular functions [50,51]. Glucose metabolism is tightly regulated through complex hormonal and metabolic pathways to maintain energy supply and systemic homeostasis. Depending on the physiological state of the body, glucose can be directed towards anabolic processes, like glycogenesis and lipogenesis, or catabolic breakdown through glycolysis [50]. Glucose homeostasis is crucial, as both hypoglycemia and hyperglycemia are associated with adverse health outcomes, which can lead to diseases and complications. Insulin and glucagon are the main hormones regulating glucose levels in circulation. Insulin promotes glucose uptake and inhibits hepatic glucose production, while glucagon counteracts hypoglycemia by stimulating glycogenolysis and gluconeogenesis, especially during fasting or stress, to maintain euglycemia [52]. Beyond its metabolic role, glucose is essential for immune cell activation and function. However, disruptions in glucose metabolism, particularly chronic hyperglycemia, can impair immune functions and contribute to metabolic disorders, such as diabetes [53]. Thus, maintaining glucose homeostasis is essential for metabolic integrity, cellular function, and immune response.

## 8. Signaling Pathways in Glucose Metabolism

Multiple pathways regulate glucose metabolism and homeostasis. The insulin-dependent phosphoinositide 3 kinase/protein kinase B (PI3K/Akt) pathway is crucial for glucose metabolism [54]. Insulin activates this pathway, leading to increased glucose uptake, glycogen and lipid synthesis, and reduced hepatic glucose production. Another important pathway is the adenosine 5′-monophosphate-activated protein kinase (AMPK), which is a master regulator of energy homeostasis [55]. AMPK is activated by cellular energy depletion and acts independently of insulin to regulate glucose metabolism in various tissues. It enhances glucose uptake and fatty acid oxidation in skeletal muscle, reduces gluconeogenesis and glycogen synthesis in hepatocytes, and inhibits lipogenesis in adipocytes.

## 9. PI3K/Akt Signaling Pathway

**Figure 2:**
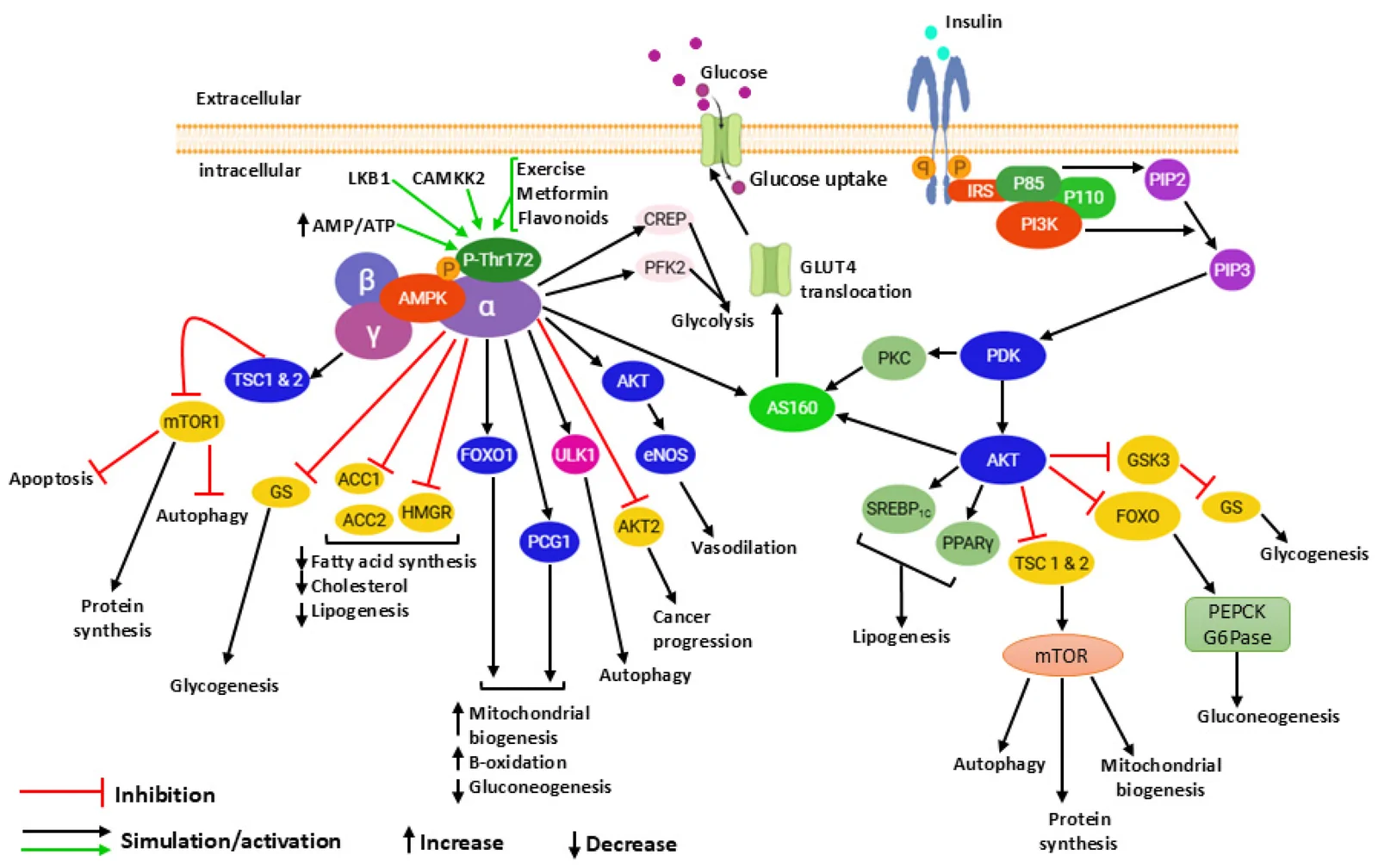
AMPK and PI3K/AKT signaling pathways involved in cardiometabolic effects.

The PI3K/Akt pathway is a pivotal molecular pathway for the metabolic actions of insulin. Insulin binds to its receptor, initiating a cascade that activates Akt and leads to downstream metabolic effects [54]. Akt activation promotes glucose uptake, inhibits glycogen synthase kinase-3β, and suppresses hepatic gluconeogenesis. The PI3K class I, specifically the p85 regulatory and p110 catalytic subunits, mediate these effects. Insulin binding to its receptor on insulin-sensitive cells triggers autophosphorylation of the receptor’s intracellular β-subunit, leading to phosphorylation of the insulin receptor substrates (IRS 1 and 2). These substrates then bind to the p85 subunit of PI3K, activating the p110 catalytic domain. Activated PI3K converts phosphatidylinositol 4,5-bisphosphate to phosphatidylinositol 3,4,5-triphosphate, which activates 3-phosphoinositide-dependent protein kinase-1 (PDK1) and ultimately leads to Akt activation (Figure 2). Akt, a serine/threonine kinase, is fully activated by phosphorylation at threonine 308 (Thr308) and serine 473 (Ser473). The activated Akt promotes glucose uptake by facilitating GLUT4 translocation and inhibits glycogen synthase kinase-3β (GSK-3β). Akt also inhibits hepatic gluconeogenesis by suppressing the transcription of phosphoenolpyruvate carboxykinase (PEPCK) and glucose-6-phosphatase through forkhead box O1 (FOXO1) inhibition.

## 10. AMPK Signaling Pathway

**Figure 3:**
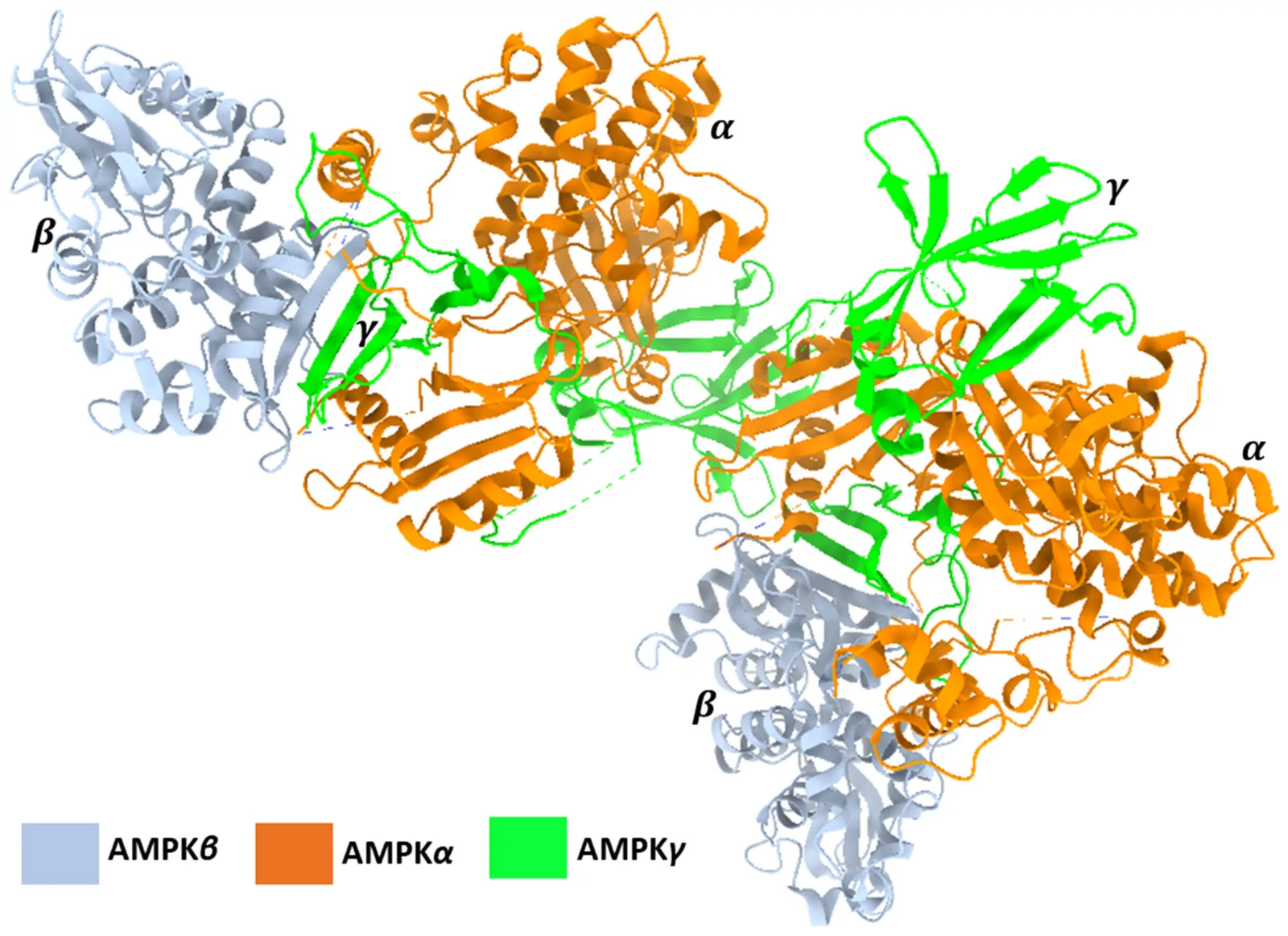
The tertiary structure of 5′ adenosine monophosphate-activated protein kinase (AMPK) showing the alpha subunit (AMPKα), beta subunit (AMPKβ), and gamma subunit (AMPKγ).

AMPK plays a key role in lipid and glucose metabolism. AMPK is a heterotrimeric complex consisting of catalytic α-subunits (α1, α2 isoforms) and regulatory β (β1, β2) and γ (γ1, γ2, γ3) subunits (Figure 3). The α1, β1, and γ1 isoforms are found in most tissues, while α2, β2, and γ2 are mainly present in skeletal and cardiac muscles [56]. AMPK activation involves phosphorylation of its catalytic α-subunit at threonine 172 (Thr172) residue by upstream kinases, such as liver kinase B1 (LKB1) and calcium/calmodulin-dependent protein kinase kinase β (CaMKKB) [57,58,59]. LKB1 activates AMPK in response to a decrease in ATP levels and a rise in AMP levels, indicating low cellular energy levels (Figure 2). CaMKKβ activates AMPK in response to elevated intracellular calcium (Ca^2+^) levels [57,58], and TAK1 activates AMPK under conditions of inflammatory stress and tumor necrosis factor (TNF). The activation of AMPK by TAK1 upregulation increases protection against apoptosis by TNF. Once activated, AMPK regulates metabolism by simulating various pathways. Activation of AMPK in skeletal muscles enhances glucose uptake by promoting the translocation of glucose transporter 4 (GLUT4) to the plasma membrane. This process involves the phosphorylation of TBC1D1, a Rab GTPase-activating protein, which facilitates GLUT4 vesicle trafficking. Additionally, AMPK stimulates fatty acid oxidation by phosphorylating and inhibiting acetyl-CoA carboxylase (ACC2), leading to decreased malonyl-CoA levels and subsequent activation of carnitine palmitoyl transferase 1 (CPT1), the rate-limiting enzyme in mitochondrial fatty acid uptake. AMPK activation in the hepatocytes suppresses gluconeogenesis by downregulating the expression of phosphoenolpyruvate carboxykinase (PEPCK) and glucose-6-phosphatase (G6Pase) [58,60,61]. This suppression is mediated through the inhibition of transcription factors, such as forkhead box O1 (FOXO1) and CREB-regulated transcription coactivator 2 (CRTC2), as well as by decreasing the activity of cAMP response element-binding protein (CREB) through glycogen synthase kinase-3β (GSK-3β) inhibition. Moreover, AMPK inhibits glycogen synthesis by phosphorylating glycogen synthase, thereby reducing its activity (Figure 2). In adipose tissues, AMPK regulates lipid metabolism. It inhibits lipogenesis by downregulating lipogenic transcription factors, such as sterol regulatory element-binding protein 1c (SREBP-1c) and carbohydrate-responsive element-binding protein (ChREBP) [58,61]. AMPK also suppresses lipolysis by phosphorylating hormone-sensitive lipase (HSL) at specific serine residues, which prevents its activation by catecholamines and subsequent translocation to lipid droplets. Furthermore, AMPK reduces triglyceride synthesis by decreasing the activity of glycerol-3-phosphate acyltransferase, an enzyme involved in triglyceride assembly.

## 11. AMPK and ACE2 Functional Integration and Their Implications for Severe Viral Infections

AMPK is widely known for its crucial role as a cellular energy sensor and coordinating metabolic stress responses. In addition to this established role, AMPK has also emerged to play a significant role in regulating vascular homeostasis, oxidative balance, and inflammatory and immune responses failure [62,63]. These expanded effects closely intersect with ACE2, a key component of the renin–angiotensin system (RAS) that regulates vascular tone and inflammatory responses, with expanding roles in metabolic and oxidative balance. Increasing evidence indicates a functional and mechanistic bridge between AMPK and ACE2 signaling, forming an integrated regulatory axis that links metabolic status to vascular and inflammatory outcomes [64]. Disruption of this coordination provides a mechanistic link on how metabolic diseases predispose to increased susceptibility to cardiovascular diseases and severe viral infections, especially in conditions, like diabetes and obesity, associated with viral infections, such as COVID-19 [65,66]. Understanding the convergence of AMPK and ACE2 signaling offers valuable insights into maintaining homeostasis and developing therapeutic interventions targeting the AMPK-ACE2 axis as a unified target that could lead to a new therapeutic strategy in addressing the intertwined pathologies of cardiometabolic and infectious diseases.

## 12. Metabolic and Physiological Roles of AMPK

AMPK plays a central role in maintaining metabolic homeostasis at cellular and systemic levels by coordinating multiple downstream pathways to elicit protective and energy-saving responses [67]. This involves regulating glucose and lipid metabolism, mitochondrial biogenesis, and autophagy failure [62,68]. When ATP is depleted and energy stress ensues, AMPK is activated to stimulate ATP-generating catabolic pathways while inhibiting energy-consuming anabolic pathways. This activation enhances insulin sensitivity, increases glucose uptake in skeletal muscle, and inhibits hepatic gluconeogenesis [68,69,70]. AMPK also contributes to maintaining systemic energy homeostasis by mediating hormonal effects in the hypothalamus, regulating appetite and energy expenditure [71,72]. AMPK enhances mitochondrial function and cellular resilience during metabolic stress by promoting mitochondrial biogenesis and reducing ROS production [73,74]. These effects are particularly beneficial in cardiomyocytes, endothelial cells, and retinal epithelial cells, enhancing their survival in high glucose or nutrient-deprived conditions. Activation of AMPK has been associated with improved metabolic health, stress resistance, and longevity, making it a promising target for therapeutic interventions in age-related diseases [61].

## 13. Role of AMPK in Cardiometabolic and Viral Pathologies

Dysregulation of AMPK has been implicated in various pathological conditions, including obesity, T2DM, NAFLD, cardiovascular diseases, and cancer [56,75]. In obesity and T2DM, reduced AMPK activity impairs fatty acid oxidation and increases lipogenesis, contributing to insulin resistance and hyperglycemia. Similarly, in diabetes, AMPK dysfunctions exacerbate hyperglycemia by enhancing gluconeogenesis. AMPK dysfunction in the heart and vasculature can contribute to the development of cardiac hypertrophy and failure [76,77]. In cancer, tumor cells impair AMPK activity to facilitate rapid growth and proliferation [78,79]. AMPK plays crucial roles in the pathogenesis of COVID-19 and diabetes, as it regulates inflammation, oxidative stress, and other cellular functions disrupted in diabetes [80,81].

## 14. AMPK Activation as a Therapeutic Target

AMPK is a pivotal regulator of cellular energy homeostasis and a therapeutic target for metabolic, cardiovascular, and inflammatory diseases. Activation of AMPK improves glucose metabolism, reduces insulin resistance in T2DM, enhances mitochondrial biogenesis, and increases expression of SIRT1 and peroxisome proliferator-activated receptor gamma coactivator-1α (PGC-1α), promoting energy expenditure [82,83]. Additionally, AMPK activation increases insulin sensitivity and GLUT4 expression, leading to improved glucose uptake in skeletal muscle [69,82]. In the liver, AMPK activation suppresses gluconeogenesis and lipogenesis while promoting fatty acid oxidation and peripheral glucose uptake, thereby mitigating hyperglycemia and dyslipidemia. AMPK provides cardiovascular protective effects by improving endothelial function through eNOS activation, reducing inflammation, and attenuating atherosclerosis [63,68]. Moreover, AMPK reduces oxidative stress, cardiac steatosis, and lipotoxicity, which are critical factors in the development of heart disease [82,84], in addition to its metabolic, cytoprotective, and anti-inflammatory effects. Its activation inhibits NF-κB signaling, suppressing inflammatory cytokine release and oxidative stress [85,86]. AMPK activation has been reported to inhibit SARS-CoV-2 replication and hyperinflammatory responses. These indicate the potential of AMPK activation in mitigating hyperinflammation, restoring glycemic control and endothelial integrity in cardiometabolic disorders and related viral pathologies. In cancer, its activation can induce apoptosis and inhibit cancer cell proliferation, indicating its potential as a tumor suppressor [78,79]. Furthermore, AMPK modulation also has relevance in neurodegenerative diseases. Its activation promotes autophagy, delays aging, and may protect against age-related diseases, like Alzheimer’s [87,88].

## 15. AMPK Activators and Therapeutic Implications

Several pharmacological and natural AMPK activators demonstrate therapeutic potential. Metformin, the most widely used antidiabetic drug, indirectly activates AMPK by inhibiting mitochondrial complex I, increasing AMP/ATP ratios. Metformin has demonstrated other benefits beyond glycemic control; it improves vascular function and reduces inflammation, making it an appealing candidate for cardiovascular and COVID-19 management [68]. Natural compounds such as resveratrol, berberine, curcumin, quercetin, and other flavonoids activate AMPK, improving glucose and lipid metabolism and reducing hepatic steatosis [89,90]. Synthetic AMPK activators, such as AICAR (5-aminoimidazole-4-carboxamide ribonucleotide) and MK8722, have demonstrated enhanced glucose uptake in skeletal muscle and improved insulin sensitivity in preclinical studies [91]. Similarly, honokiol, a biphenolic compound, demonstrated AMPK-mediated metabolic improvements in models of T2DM and NAFLD [70,82]. Interestingly, AMPK activation also plays a role in viral pathophysiology, bridging metabolic and viral pathways. Restoration of AMPK activity led to reduced HCV replication and lipid accumulation [23]. Metformin and flavonoids not only activate AMPK but also modulate ACE2 expression, which may confer endothelial protection in cardiometabolic disorders and COVID-19 [92]. This dual effect suggests potential synergistic benefits between AMPK activators and ACE2 modulators, mitigating cardiometabolic and infectious complications. Physiologically, exercise and caloric restriction also activate AMPK by reducing ATP levels and increasing AMP, which promotes catabolic pathways and inhibits anabolic pathways [93]. Tissue-specific effects of AMPK also enhance its therapeutic potential for a targeted therapy that could maximize benefits with minimal side effects. AMPK activation in the liver inhibits gluconeogenesis and lipogenesis, while in the hypothalamus, it reduces appetite and increases energy expenditure [94]. The AMPK signaling pathway represents a promising target for various diseases, including T2DM, NAFLD, cardiovascular, neurodegenerative, and infectious diseases [89,95].

## 16. ACE2 and the Renin–Angiotensin System (RAS)

**Figure 4:**
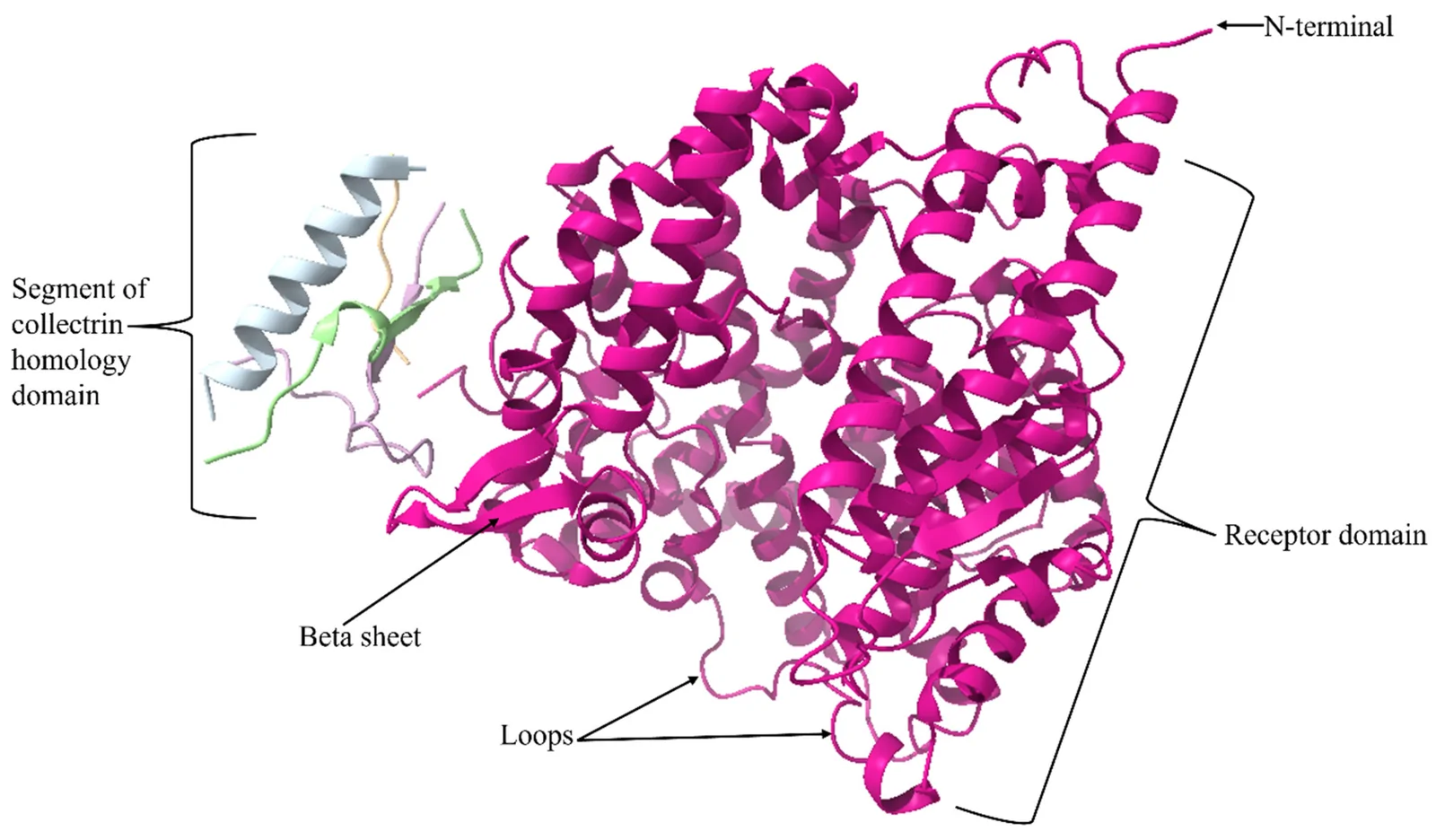
The 3D structure of angiotensin-converting enzyme 2 (ACE2).

Angiotensin-converting enzyme 2 (ACE2) is a transmembrane glycoprotein and zinc metallopeptidase (Figure 4) that plays a crucial role in maintaining cardiovascular, renal, pulmonary, and metabolic homeostasis [64]. It is a functional homolog of angiotensin-converting enzyme (ACE) but exerts opposing effects within the renin–angiotensin system (RAS).

**Figure 5:**
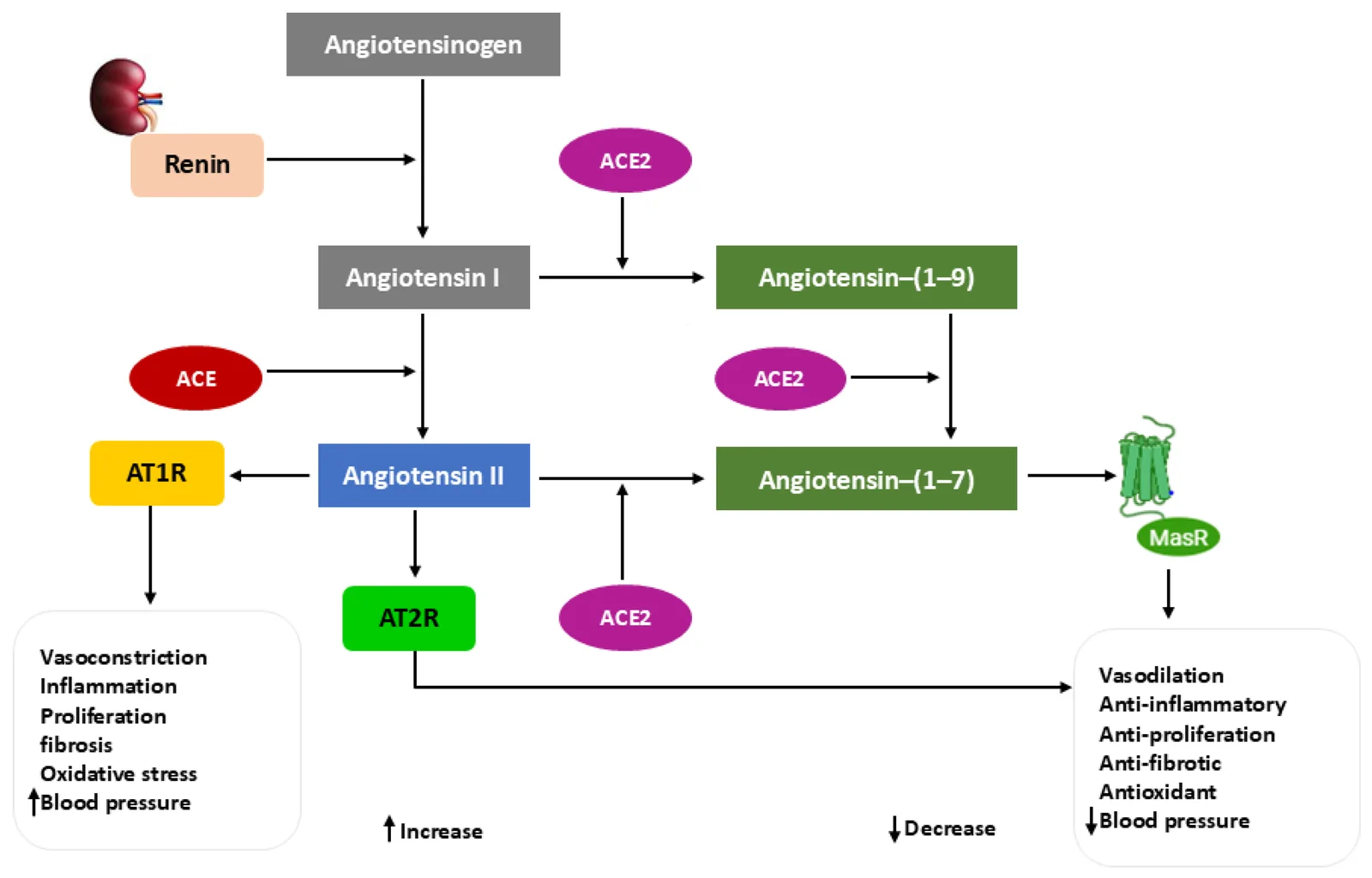
Counter-catalytic roles of ACE2 in the renin–angiotensin system (RAS).

ACE cleaves angiotensin I (Ang I) into angiotensin II (Ang II), a potent vasoconstrictor that promotes inflammation, oxidative stress, and fibrosis, while ACE2 hydrolyzes Ang II into angiotensin-(1–7) (Ang-(1–7)), activating the Mas receptor to elicit vasodilatory, anti-inflammatory, and antifibrotic responses (Figure 5), against the harmful Ang II/AT1 receptor axis [96,97].

ACE2 is expressed in endothelial and epithelial cells of the lungs, heart, kidneys, pancreas, intestine, adipose tissue, and vasculature, contributing to blood pressure regulation, electrolyte balance, vascular tone, and metabolism [96,98]. In addition to angiotensin peptides, ACE2 metabolizes other substrates, such as apelin, neurotensin, dynorphin A1–13, casomorphin, and kinetensin, linking it to neurohumoral and metabolic regulation. Experimental evidence further suggests that ACE2 modulates sympathetic outflow and neurohumoral control of blood pressure, reinforcing its systemic regulatory capacity [96,99]. In cardiovascular and pulmonary physiology, the ACE2/Ang 1–7/MasR axis has protective effects, improving endothelial function, attenuating oxidative stress, and restricting vascular remodeling [96,99,100]. ACE2 plays a critical role in maintaining cardiovascular, metabolic, and pulmonary homeostasis but also serves as the major entry receptor for SARS-CoV-2 [101].

## 17. ACE2 in Metabolic and Cardiovascular Regulations

**Figure 6:**
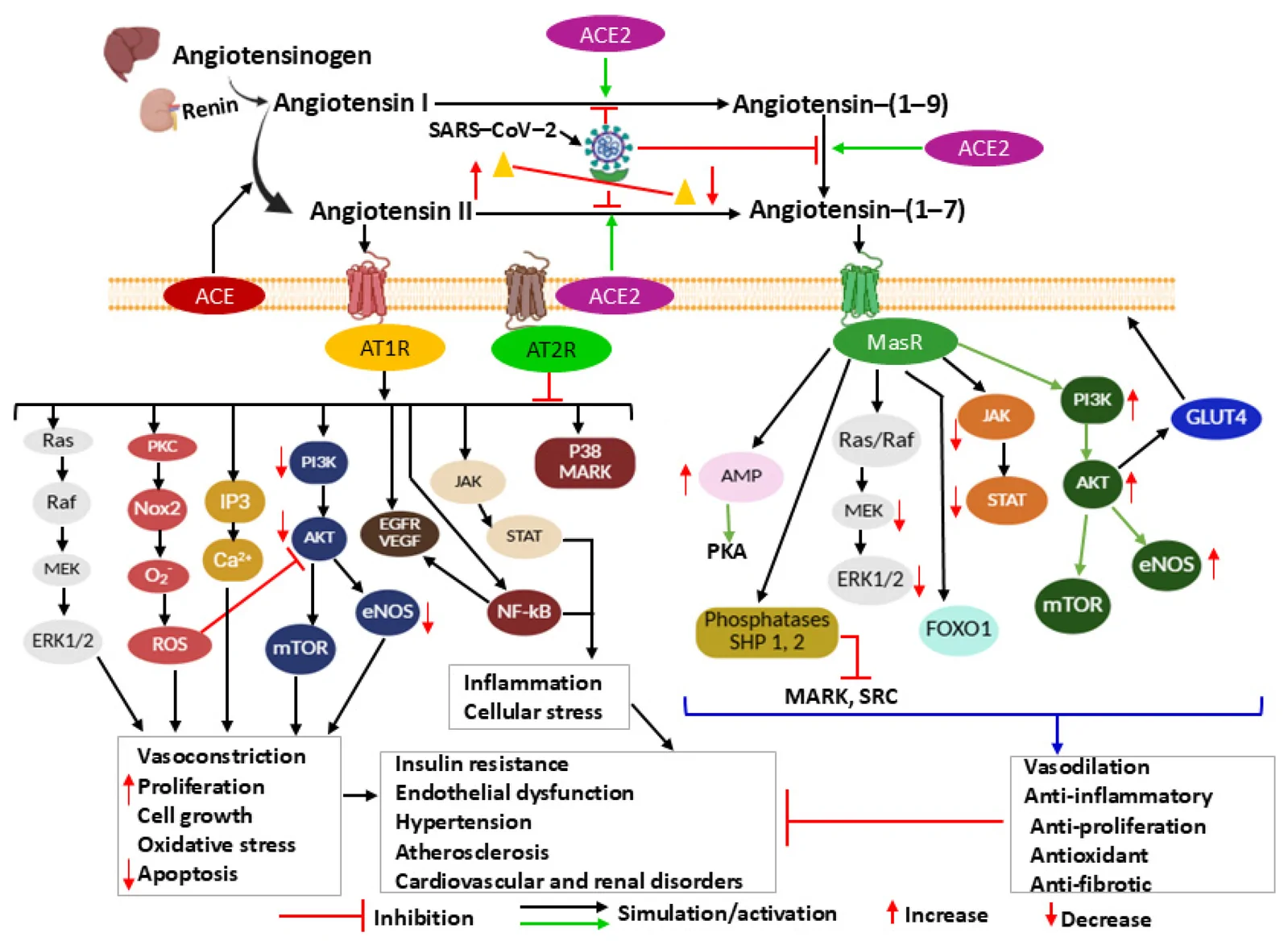
Downstream receptor activation and signaling pathways of ACE and ACE2 with SARS-CoV-2 signal disruption.

ACE2 plays a multifaceted role in maintaining metabolic and cardiovascular homeostasis by enhancing insulin sensitivity and glucose metabolism and regulating the renin–angiotensin system. This offers potential therapeutic strategies for metabolic disorders, including type 2 diabetes [102]. ACE2 plays a critical role in pulmonary vascular health, specifically in promoting vasodilation and countering fibrosis and hypertrophy [103,104]. ACE2 promotes vasodilation, reduces inflammation, and inhibits fibrosis in the cardiovascular system [105,106]. ACE2 has also been shown to decrease the expression of pro-inflammatory cytokines and improve endothelial function, which is important for preventing cardiovascular disease [65,107]. ACE2 reduces blood pressure and inflammation, protects the lung against injury and acute respiratory distress syndrome (ARDS) [108], and is essential in the regulation of glucose homeostasis [109]. Decreased ACE2 expression in obesity leads to inflammation and insulin resistance, key components of metabolic syndrome [110]. Studies show that ACE2 deficiency aggravates cardiac dysfunction and metabolic abnormalities in obese mice, while increased ACE2 expression improves glucose tolerance and reduces inflammation [111,112]. ACE2 is involved in regulating energy metabolism and thermogenesis [113]. ACE2 deficiency has been associated with impaired thermogenesis and metabolic dysfunction, underscoring its significance in maintaining metabolic homeostasis [111,114]. ACE2 plays a critical role in maintaining metabolic balance and insulin sensitivity by regulating GLUT4 expression in skeletal muscle. ACE2 knockout mice exhibited decreased GLUT4 expression and impaired glucose uptake in skeletal muscle in the absence of ACE2 [109]. However, administration of Ang-(1–7), a product by ACE2, restores GLUT4 levels and improves insulin sensitivity [109,111]. ACE2 activation enhances AMPK and endothelial nitric oxide synthase (eNOS) signaling, improving insulin sensitivity and glucose metabolism [83]. ACE2 also preserves pancreatic β-cell function and glucose homeostasis, with ACE2 overexpression promoting β-cell proliferation and reducing apoptosis [115]. Studies have reported that ACE2 modulates glucose metabolism through gamma-aminobutyric acid (GABA) signaling in pancreatic β-cells [116,117]. Maintaining the balance between ACE and ACE2 enzymes is vital for proper organ functions and cardiovascular health [99], and any disruption can result in disease pathogenesis or worsening of existing conditions (Figure 6).

## 18. Role of ACE2 in Inflammation and Immunological Modulation

ACE2 modulates inflammation through the Ang II/Ang-(1–7) axis. A decrease or depletion of ACE2 shifts the equilibrium towards Ang II predominance, leading to the production of pro-inflammatory cytokines, like IL-6 and TNF-α [16,65]. This inflammatory response exacerbates insulin resistance and lipid dysregulation, creating a feedback loop between metabolic dysfunction and systemic inflammation. The downregulation of ACE2 fuses the intersection of metabolic and inflammatory pathways, worsening these effects, particularly in individuals with pre-existing metabolic disorders, leading to poorer outcomes [66,118]. Additionally, adipokines like leptin and adiponectin, disrupted in obesity and diabetes, interact with ACE2-mediated pathways. Reduced ACE2 activity can decrease adiponectin levels, which protect against inflammation and insulin resistance while increasing leptin resistance, promoting inflammation and metabolic dysfunction (Figure 6).

## 19. ACE2 and Its Implications in Disease Pathogenesis

ACE2 plays a significant role in regulating the renin–angiotensin system (RAS) and maintaining vascular, renal, and metabolic homeostasis (Figure 5). Dysregulation of ACE2 can lead to various diseases by disrupting the balance between angiotensin II (Ang II) and angiotensin-(1–7) (Ang-(1–7) [119]. Reduced ACE2 activity results in decreased Ang-(1–7) formation and increased Ang II accumulation, leading to harmful effects such as vasoconstriction, oxidative stress, inflammation, fibrosis, and endothelial dysfunction [100,107,120]. Conversely, Ang-(1–7) exerts beneficial effects, including vasodilation, anti-inflammatory properties, and anti-fibrotic effects (Figure 5 and Figure 6), indicating the importance of ACE2 in maintaining vascular integrity and homeostasis [99].

Maintaining an optimal balance between ACE and ACE2 is essential for cardiovascular and metabolic health. Dysregulation of this equilibrium has been associated with conditions like hypertension, atherosclerosis, heart failure, chronic kidney disease, and metabolic disorders, mainly due to excessive Ang II signaling and RAS overactivation [64,99]. Experimental studies from models of ischemia, myocardial infarction (MI), and hypertension have shown that ACE2 deficiency or deletion leads to RAS hyperactivation, oxidative tissue damage, cardiac fibrosis, and dysfunction [121,122]. Similarly, ACE2 loss has also been linked to neurogenic hypertension, early cardiac hypertrophy, increased pro-inflammatory cytokines, and activation of ERK1/2 and JNK1/2 signaling pathways in peri-infarct regions after MI [111,123]. In diabetes, ACE2 deficiency worsens vascular dysfunction, diabetic nephropathy, and retinopathy [124,125]. Moreover, ACE2 deficiency exacerbates acute respiratory distress syndrome (ARDS), hypertension, thrombosis, myocardial injury, and acute kidney injury (AKI) [66,126].

Furthermore, ACE2 also serves as the primary cellular entry receptor for SARS-CoV-2 [101]. Viral binding downregulates ACE2, disrupting the ACE2/Ang 1–7/MasR axis and RAS equilibrium. This disruption leads to increased Ang II-mediated effects, like vasoconstriction, inflammation, and fibrosis, which are significant in COVID-19 pathology [127,128]. The consequent dysregulation of ACE2 contributes to endothelial dysfunction, cytokine release, oxidative stress, thrombosis, and insulin resistance, potentially explaining the increased severity of COVID-19 observed in patients with metabolic comorbidities [97,129]. ACE2 dysregulation has also been implicated in obesity, diabetes, and metabolic syndrome, stressing its role in glucose regulation, lipid metabolism, and energy homeostasis [120,127].

ACE2 protects against vascular and cardiopulmonary injury by converting Ang I and Ang II into Ang-(1–9) and Ang-(1–7), respectively, counteracting the detrimental effects of Ang II signaling [130]. In the heart, ACE2 helps preserve myocardial contractility and prevent maladaptive hypertrophy and fibrosis, while in the lungs, it attenuates Ang II-induced inflammation, fibrotic injury, and vascular leakage [64,97,126]. However, despite these protective effects, the explicit downstream metabolic signaling pathways modulated by ACE2 are not fully understood. ACE2 represents a central molecular node connecting cardiovascular, renal, metabolic, and infectious disease pathways [131]. Its dual roles, as a protective RAS regulator and a viral receptor for SARS-CoV-2, spark its significance as a therapeutic target. Hence, strategies aimed at restoring ACE2 activity or enhancing the Ang-(1–7)/Mas receptor axis may offer substantial interventions for mitigating vascular, pulmonary, and metabolic complications associated with COVID-19 and related cardiometabolic disorders.

## 20. ACE2/Ang-(1–7)/Mas Receptor Axis as a Therapeutic Target

ACE2 exerts its physiological effects primarily through the ACE2/Ang-(1–7)/Mas receptor axis. It elicits vasodilatory, anti-inflammatory, and metabolic effects to maintain cardiovascular and metabolic balance [132]. Activation of this pathway stimulates multiple downstream pathways to mitigate the deleterious effects of the Ang II/AT1R pathway (Figure 6). The cardiovascular and metabolic benefits of this signaling pathway have increasingly been recognized as a therapeutic target for cardiometabolic disorders.

Therapeutic activation of ACE2 signaling through Ang-(1–7), recombinant human ACE2 (rhACE2), or genetic expression has consistently demonstrated benefits in several preclinical models. Small molecules and ACE2 activators, such as diminazene aceturate, elevated Ang-(1–7) levels and improved metabolic outcomes in experimental models [133], indicating potential therapeutic benefits of ACE2 activation in metabolic diseases. ACE2 activation or increased expression enhanced insulin sensitivity and metabolic homeostasis by reducing inflammation and improving lipid metabolism [111]. Treatment with Ang-(1–7) or rhACE2 ameliorated cardiac steatosis and lipotoxicity and improved cardiovascular remodeling in animal models, reflecting the therapeutic implications in diabetes and cardiac dysfunction. Similarly, ACE2 activation reduces lipogenesis while promoting fatty acid oxidation and thermogenesis, which enhances energy expenditure and metabolic functions [65,113]. Further studies reported that therapeutic activation of ACE2 showed therapeutic promise in mitigating inflammation, cardiovascular, and metabolic dysfunctions in preclinical models [65,121]. These studies provide compelling evidence that ACE2 activation attenuates lipotoxic events and preserves cardiac and metabolic functions. ACE2 activation also modulates various intracellular pathways that are crucial for mitochondrial and metabolic homeostasis. Ang-(1–7) activation of Mas receptor stimulates Akt/FOXO1 and PKA signaling, leading to increased UCP1 expression and improved mitochondrial function [113], linking ACE2 signaling and mitochondrial bioenergetics. Conversely, ACE2 signaling was shown to inhibit the p38 MAPK pathway, attenuating inflammation and improving insulin sensitivity [83,104,111]. This reinforces the metabolic and anti-inflammatory role of the ACE2/Ang-(1–7)/Mas receptor axis. Studies continued to elucidate the cardiometabolic benefits and protective effects of ACE2 signaling. Administration of recombinant human ACE2 (rhACE2) was reported to decrease ANG II-mediated cardiac hypertrophy, fibrosis, and dysfunction [134,135], while increased expression of ACE2 or Ang-(1–7) treatment has been shown to improve cardiac remodeling, lower blood pressure, and mitigate cardiomyopathy, atherosclerosis, and diabetic nephropathy [136,137]. These suggest that ACE2 exerts a broad protective and beneficial role across several organ systems vulnerable to metabolic, renal, and vascular injury.

Moreover, recent emerging findings uncovered a neuroendocrine dimension to ACE2 signaling. Activation of ACE2 also enhances GABA expression and β-cell function, thereby improving glucose metabolism and energy homeostasis [116,117]. This suggests that ACE2 also modulates central regulatory mechanisms that coordinate energy balance, which may have implications for diabetes and other metabolic disorders. Understanding ACE2-related signaling networks and investigating ACE2 modulators could unveil a potential strategy against cardiovascular, metabolic, neurodegenerative, and viral diseases, particularly in conditions like COVID-19, where ACE2 serves as a viral entry receptor and a regulator of inflammation, vascular tone, and metabolism [138,139]. Thus, integrated approaches that bridge cardiovascular, metabolic, and neurological benefits offer a panacea for cardiometabolic and viral infectious diseases.

## 21. Overview of COVID-19 Pathogenesis and Complications

**Figure 7:**
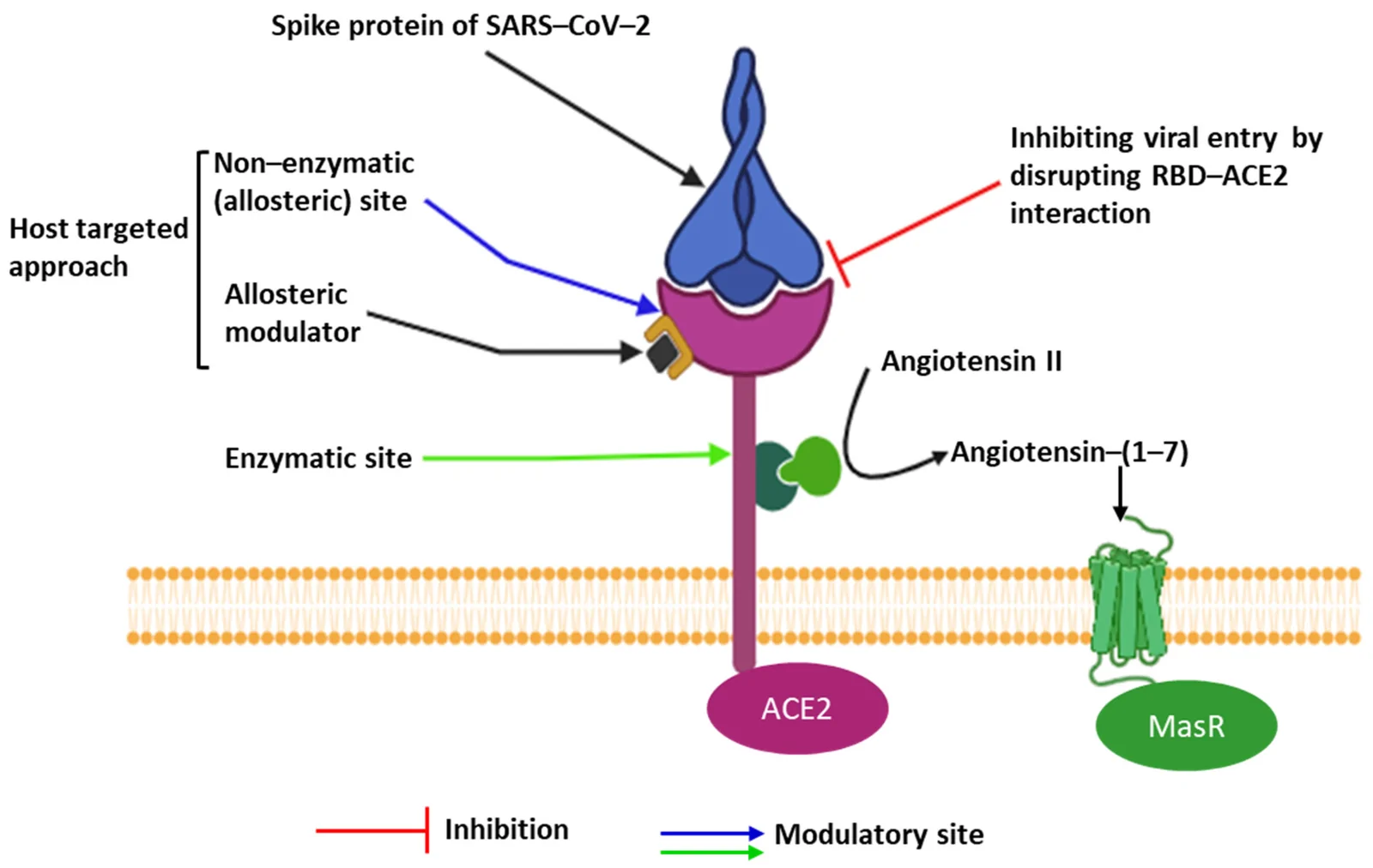
ACE2 interaction with the spike protein of SARS-CoV-2 with potential therapeutic targets.

The pathogenesis of COVID-19 involves multifaceted mechanisms, including viral invasion, host cell replication, immune dysregulation, renin–angiotensin system (RAS) imbalance, oxidative stress, and endothelial dysfunction [140,141]. These mechanisms drive inflammation, respiratory impairment, and multi-organ complications, thereby increasing morbidity and mortality rates [142,143]. COVID-19 severity arises from the interplay of viral factors, host immune responses, RAS imbalance, and endothelial dysfunction. Understanding these interconnected mechanisms is essential for guiding therapeutic interventions and improving patient outcomes. SARS-CoV-2 primarily infects host cells by binding to the angiotensin-converting enzyme 2 (ACE2) receptor through its spike (S) protein (Figure 7). This interaction not only enables viral entry but also downregulates ACE2, disrupting the RAS homeostasis (Figure 6). Consequently, elevated angiotensin II levels promote vasoconstriction, vascular permeability, and widespread inflammation, leading to respiratory and cardiovascular complications [16]. Additionally, viral infection and host immune activation induce the generation of reactive oxygen species (ROS), resulting in oxidative stress and damage to cellular components such as DNA, lipids, and proteins. Moreover, the virus disrupts mitochondrial function, reducing energy production and exacerbating cellular stress and apoptosis. These metabolic disruptions contribute to organ injury and disease progression [142,144,145].

The severity of COVID-19 is influenced by the host’s immune response. In mild cases, effective antiviral responses eliminate the virus. However, in severe cases, SARS-CoV-2 evades the early immune response by suppressing interferon production, delaying viral clearance, and facilitating viral replication [146]. Subsequent immune hyperactivation or dysregulated immune response with elevated cytokine levels and T-cell depletion leads to systemic inflammation, ARDS, and multi-organ damage [143,144]. Inflammatory mediators from immune cells, particularly alveolar macrophages, contribute to lung injury and fibrosis. Infection of endothelial cells by the virus induces endothelial dysfunction and injury, vascular inflammation, and coagulopathy [147,148]. This exacerbates lung injury and fibrosis and increases the risk of thrombosis, pulmonary embolism, and multi-organ damage, especially in individuals with pre-existing hypertension [141,149]. Viral mechanisms and host factors impact pathogenicity and disease severity. Advanced age, genetic predispositions, and comorbidities, such as diabetes, obesity, and hypertension, amplify systemic inflammation and worsen outcomes [142,143]. Viral mutations, particularly within the spike protein, further modulate transmissibility, immune evasion, and vaccine effectiveness, complicating disease management and therapeutic strategies. The convergence of RAS imbalance, oxidative stress, immune dysregulation, and endothelial injury leads to severe pneumonia, ARDS, sepsis, and multi-organ damage [150,151]. These mechanisms highlight the complexity of COVID-19 pathophysiology and underscore the importance of therapeutic strategies targeting not only viral replication but also host inflammatory, oxidative, and vascular pathways.

## 22. Comorbidities and the Relative Risks of Severe COVID-19

Diabetes, hypertension, obesity, chronic kidney disease (CKD), and other related cardiometabolic conditions are the most significant comorbidities and risk factors contributing to severe COVID-19, each amplifying susceptibility through interconnected mechanisms of metabolic dysfunction, inflammation, and endothelial impairment. Patients with these comorbidities have consistently shown greater susceptibility to developing complications, poorer treatment outcomes, prolonged hospitalizations, and higher mortality rates [152,153].

Diabetes exacerbates COVID-19 severity through hyperglycemia, insulin resistance, and systemic inflammation [154]. Poor glycemic control in diabetes is linked to higher mortality (OR: 1.18, 95% CI: 1.09–1.27) [155]. Hyperglycemia impairs immune responses and enhances pro-inflammatory cytokine release, predisposing to cytokine storm, a hallmark of severe COVID-19 [156]. Similarly, diabetic patients with renal complications have a higher risk for infection, hospitalization, and adverse outcomes [157]. Hypertension is another critical risk factor, which predisposes patients to severe COVID-19, with patients showing a higher risk of invasive mechanical ventilation (OR: 1.696) [14,158]. Hypertension linked to adverse COVID-19 outcomes contributes to disease severity by promoting endothelial dysfunction, increasing vascular permeability, pulmonary inflammation, and oedema, leading to respiratory failure. Genetic studies further suggested overlapping inflammatory and immune-related pathways between hypertension and COVID-19 severity [159]. Obesity is another independent risk factor, particularly in class III obesity, which elevates the need for mechanical ventilation and mortality [160,161]. Excess adiposity promotes chronic low-grade inflammation and impairs immune responses, amplifying viral pathogenesis and the risk of severe COVID-19. Mechanical factors associated with excess body fat reduce lung capacity, further increasing the risk of respiratory failure and the need for mechanical ventilation in obese individuals [152,162,163]. These metabolic conditions predispose individuals to complications such as acute kidney injury, acute respiratory distress syndrome (ARD), sepsis, shock, and multi-organ failure [164].

Cardiovascular and renal diseases, including coronary artery disease, heart failure, and chronic kidney disease (CKD), elevate COVID-19 severity through RAS dysregulation, oxidative stress, immune suppression, systemic inflammation, and endothelial dysfunction [165,166]. CKD patients are particularly vulnerable to acute kidney injury, sepsis, prolonged hospitalization, and higher mortality [164,166]. These conditions often comorbid with metabolic disorders, compounding immunoinflammatory and vascular dysfunction [167]. These comorbidities share overlapping pathophysiological pathways, chronic inflammation, immune dysregulation, oxidative stress, and endothelial dysfunction that synergize to exacerbate the clinical course of SARS-CoV-2 infection, highlighting the importance of addressing these vulnerabilities to improve outcomes in patients with metabolic comorbidities. Optimizing metabolic and cardiovascular management may, therefore, mitigate COVID-19 severity and improve clinical outcomes in high-risk populations.

## 23. Interplay of ACE2 and AMPK Pathways and Their Therapeutic Implications

**Figure 8:**
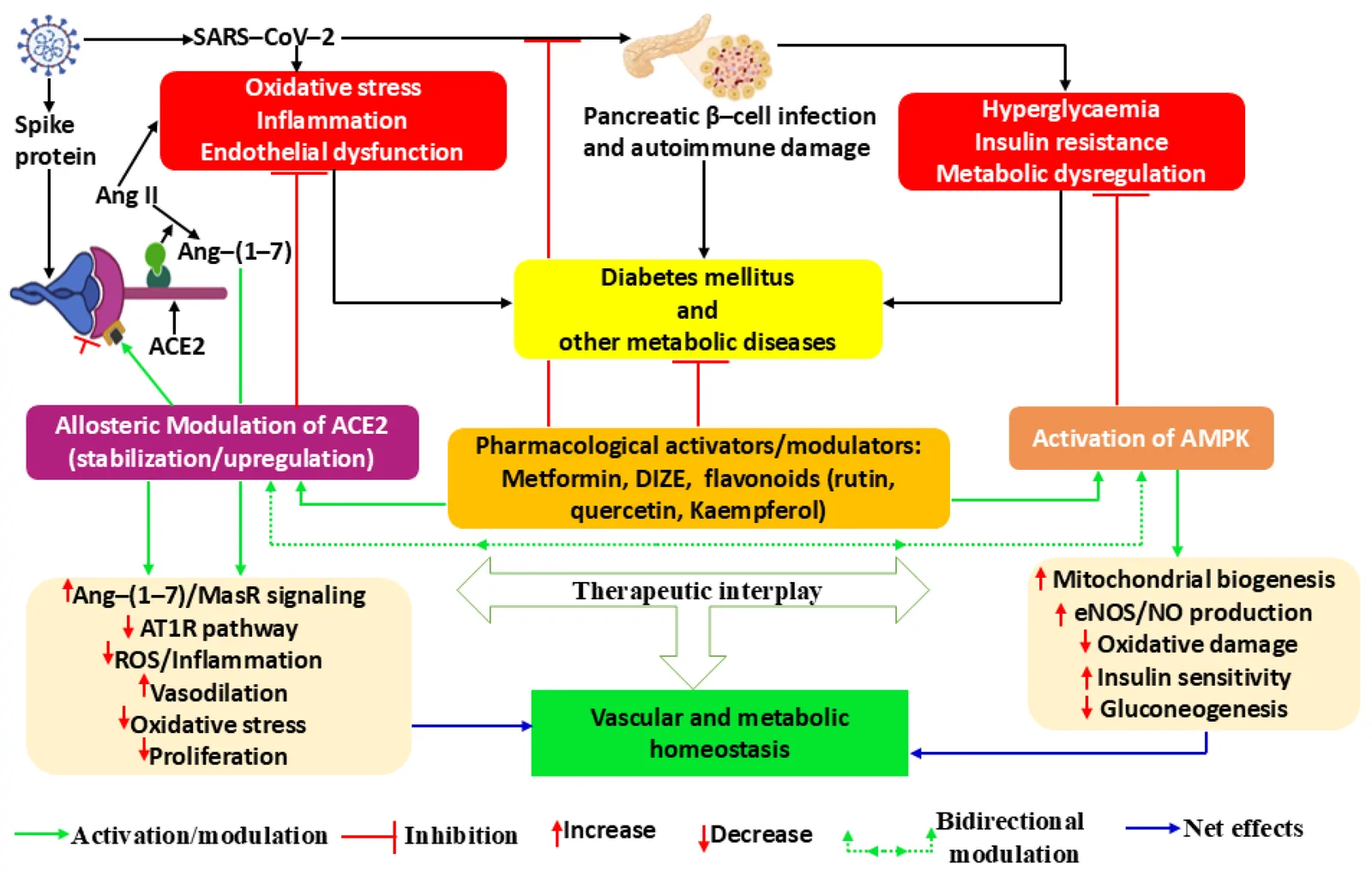
Potential benefits of dual targeting of the AMPK-ACE2 axis for mitigating cardiometabolic diseases and COVID-19-related metabolic complications.

The interplay between AMPK and ACE2 pathways represents a promising therapeutic axis in cardiovascular, metabolic, and pulmonary diseases (Figure 8). AMPK, a master regulator of cellular energy homeostasis, can modulate ACE2 expression and stability, thereby influencing vasodilation, inflammation, and oxidative stress [168,169]. ACE2, a critical component of the ACE2/Ang-(1–7)/Mas receptor axis, plays a significant role in renal and cardiovascular health but also serves as a SARS-CoV-2 entry receptor, linking it to the post-COVID-19 cardiometabolic dysfunction (Figure 7 and Figure 8). AMPK phosphorylates ACE2 at Ser680, enhancing its stability and promoting the conversion of angiotensin II (Ang II) to angiotensin-(1–7) [103]. This activates endothelial nitric oxide synthase (eNOS) and increases nitric oxide (NO) bioavailability, which improves vasodilation and attenuates pulmonary hypertension (PH) and fibrosis. These effects were demonstrated in monocrotaline (MCT) induced PH in rats and SU5416 hypoxia/reoxygenation in mice, where AMPK-mediated ACE2 phosphorylation improved endothelial function while ACE2 S680D knock-in mice showed reduced right ventricular systolic pressure (RVSP), Fulton index, pulmonary artery muscularization, and luminal narrowing [103]. These studies highlight the therapeutic potential of coactivating AMPK and ACE2 to enhance endothelial function and vascular homeostasis.

Activation of the ACE2/Ang-(1–7)/Mas axis reduces inflammation and improves vascular function in lung and cardiovascular diseases. AMPK activation also enhances insulin sensitivity, mitochondrial function, glucose uptake, and lipid metabolism, reinforcing its therapeutic role in type 2 diabetes and metabolic syndrome [82,113].

Ashkar et al. (2023) reported that the bioactive peptide IRW upregulated ACE2 expression, activating AMPK and increasing glucose transport 4 (GLUT4) expression, thereby improving glucose metabolism and insulin responsiveness [83]. This dual regulatory mechanism suggests that targeting the ACE2-AMPK signaling pathway could mitigate insulin resistance while enhancing vascular health, indicating the potential of IRW in managing metabolic complications. The study by Chen et al. (2024) demonstrated that Przewaquinone A (PA) from Salvia przewalskii Maxim exhibited protective effects against endothelial dysfunction in human umbilical vein endothelial cells (HUVECs) and Ang II-induced hypertensive mice by activating AMPKα, enhancing eNOS-mediated NO release, and reducing endothelin-1 (ET-1) levels, leading to improved vascular function and lowered blood pressure [170]. The AMPKα activation-dependent effects of PA highlight the synergy of AMPK-ACE2 signaling and offer a novel therapeutic avenue for both metabolic and cardiovascular benefits. These findings highlight the synergy of AMPK-ACE2 signaling in vascular and metabolic regulation. Mondal et al. (2024) found that hyperglycemia-induced yes-associated protein 1 (YAP1) overexpression suppressed ACE2, upregulated β-catenin and angiotensin-converting enzyme (ACE), and activated TGFB1/SMAD2–3 signaling, driving cardiac hypertrophy and fibrosis [171]. This reinforces the protective role of ACE2 in diabetic cardiomyopathy and suggests that AMPK-ACE2 targeting could prevent hyperglycemia-related cardiovascular complications. Odaira-Satoh et al. (2025) reported that captopril and Ang-(1–7) exerted antidepressant effects in olfactory bulbectomized (OBX) mice by enhancing hippocampal neurogenesis through hippocampal ACE2/Ang (1–7)/MasR/AMPK/BDNF signaling [172], indicating that ACE2-AMPK modulation extends to neurological health.

Targeting AMPK-ACE2 signaling could offer dual benefits by restoring renovascular and metabolic homeostasis [173]. These strategies may be particularly valuable in COVID-19 cardiometabolic complications, where both pathways modulate inflammation, mitochondrial function, and vascular health (Figure 8). AMPK activators, such as metformin and berberine, along with ACE2 modulators, may yield additive or synergistic benefits in vasodilation, metabolic regulation, and anti-inflammatory responses and are promising candidates for combined interventions. Despite encouraging preclinical evidence, most studies investigated AMPK or ACE2 independently. The coordinated regulation of AMPK and ACE2 in cardiometabolic and pulmonary diseases remains underexplored. Furthermore, the balance between synergistic and antagonistic effects of dual targeting is critical and poorly understood. Hence, further research is required to explore their bidirectional therapeutic mechanisms in diabetes and cardiovascular disorders, which often coexist as comorbidities and are responsible for most cases of COVID-19 severity and mortality.

The strength of evidence supporting pharmacological modulators of AMPK and ACE2 varies among different classes of bioactive compounds. Metformin, a well-studied AMPK activator, has substantial experimental and clinical data demonstrating its metabolic and cardiovascular benefits [103,174]. However, direct clinical evidence connecting metformin-mediated AMPK activation to ACE2 modulation is lacking. Flavonoids have been reported to modulate AMPK signaling and have limited reports on ACE2 activity. However, most of the available evidence is from in vitro and animal studies, or computational analyses with limited clinical testing. Similarly, pharmacological agents targeting ACE2 or the ACE2/Ang-(1–7)/Mas axis, such as diminazene aceturate (DIZE) or Ang-(1–7), have shown promising effects in preclinical models but lack robust clinical evidence [133]. Hence, understanding these differences in evidence is essential for effectively assessing the therapeutic potential of targeting the AMPK-ACE2 axis.

Targeting the AMPK-ACE2 axis presents significant therapeutic potential but also challenges. AMPK and ACE2 have tissue-specific regulatory patterns, resulting in varied metabolic and cardiovascular responses upon activation [62,64]. Hence, a thorough evaluation of the potential risks and systemic outcomes is necessary to assess off-target effects when targeting this axis. Additionally, modulating ACE2, a cellular entry receptor for SARS-CoV-2, requires considering its potential effects on viral entry dynamics during infection. Furthermore, experimental evidence supporting the metabolic and vascular effects of concurrently activating AMPK and ACE2 signaling is still limited, and translation into clinical evaluation remains unexplored, as most candidate modulators have mainly been studied only in vitro, in animal models, or through computational methods. Consequently, well-designed clinical trials are required to evaluate their safety and effectiveness. Thus, overcoming these challenges is crucial for advancing the AMPK-ACE2 axis from a mechanistic concept into a viable therapeutic strategy and clinical application.

## 24. Flavonoids

Flavonoids are a diverse group of bioactive polyphenols found in natural products and are known for their antioxidant, anti-inflammatory, antidiabetic, and cardiometabolic protective properties (Table 1). These effects are mediated through modulation of redox balance, inflammatory signaling, and metabolic pathways [175,176]. There is substantial evidence supporting the potential of flavonoids in preventing and treating chronic diseases related to oxidative stress, inflammation, and metabolic dysfunction [177,178]. AMPK is a key molecular target of flavonoids, with both direct and indirect activation contributing to improved glucose and lipid metabolism, enhanced insulin sensitivity, mitochondrial function, and endothelial integrity [89,179]. Several flavonoids, such as rutin, apigenin, kempferol, quercetin, luteolin, myricetin, isorhamnetin, and resveratrol, activate AMPK directly or indirectly, improve insulin sensitivity, glucose, and lipid metabolism, restrain inflammatory signaling, and prevent cellular oxidative damage, leading to metabolic regulation, neuroprotection, cardioprotective effects, systemic homeostasis, and metabolic health [180,181,182,183,184,185,186]. By inhibiting lipogenesis, promoting fatty acid oxidation, and attenuating oxidative stress and inflammation, these compounds offer broad pharmacological benefits in various cardiometabolic diseases [187,188]. In addition to metabolic regulation, flavonoids have demonstrated immunomodulatory properties relevant to viral pathogenesis. Metabolic diseases such as diabetes, obesity, and NAFLD amplify viral disease severity through chronic inflammation, oxidative stress, and endothelial dysfunction [189,190]. Flavonoids mediated AMPK activation and regulation of these pathways can mitigate these processes and suppress pro-inflammatory cytokines, such as TNF-α, IL-6, and IL-1β, mitigating cardiometabolic dysfunction and severe viral immunopathology [191,192]. The therapeutic potential of flavonoids in managing metabolic diseases and inflammatory complications while enhancing resilience to severe viral infections is promising. The therapeutic benefits of compounds from natural products have been widely reported [193,194]. Emerging evidence suggests that flavonoids may also influence ACE2, a key regulator of vascular homeostasis and SARS-CoV-2 cellular entry. However, agents capable of modulating ACE2 activity or its expression, as well as the understanding of their mechanisms of action, remain limited.

Several flavonoids exhibit overlapping (Table 1) yet distinct mechanisms of action. Apigenin, rutin, and other flavonoids by targeting AMPK, oxidative stress, and inflammatory pathways improved insulin sensitivity and glucose metabolism, exerted cardioprotective and antidiabetic effects [180,181,195], enhanced mitochondrial function and lipid metabolism [182,196], and regulated redox-sensitive, immune, and metabolic signaling [184,185,197]. Quercetin regulates several pathways with significant pharmacological properties, including increased insulin responsiveness, vascular function, and modulated immune mediators, reinforcing flavonoid therapeutic pleiotropy [183,198], while resveratrol, a potent activator of AMPK, exhibits pronounced cardioprotective and neuroprotective effects [186]. Studies suggest that several flavonoids may modulate ACE2 expression or activity, potentially modulating the AMPK-ACE2 axis. Flavonoids such as quercetin and luteolin have shown interactions with ACE2 in both experimental and computational studies [198,199], suggesting potential effects on the viral entry mechanism associated with SARS-CoV-2 and the renin–angiotensin system. Similarly, hesperidin and rutin have been reported to potentially interact with ACE2 through molecular docking and network pharmacology analyses. However, most evidence regarding the effects of flavonoids on ACE2 functions is limited to in silico predictions and experimental preclinical studies, and the exact molecular mechanisms by which flavonoids modulate ACE2 are not adequately and fully elucidated. Consequently, further experimental and clinical studies are required to establish the ACE2-modulating effects of these compounds. These diverse pharmacological effects are particularly relevant in severe viral infections, where metabolic dysregulation and hyperinflammation synergistically drive susceptibility to viral infections and disease progression in metabolically compromised individuals.

Despite compelling preclinical evidence, critical gaps remain regarding the binding affinities, tissue-specific signaling of flavonoids, and their coordinated modulation of AMPK and ACE2 in complex disease states. Metformin, a known antidiabetic and indirect AMPK activator, has been reported to exert anti-inflammatory effects and modulate ACE2 expression and function, suggesting a potential therapeutic role in COVID-19 [200,201]. Flavonoids represent promising leads for a multifunctional therapeutic approach that may limit and mitigate metabolic, vascular, and immune-related pathologies while enhancing host resilience against severe viral infections through a coordinated modulation of AMPK pathways, suppressing inflammatory signaling and ACE2-associated mechanisms.

**Figure 9:**
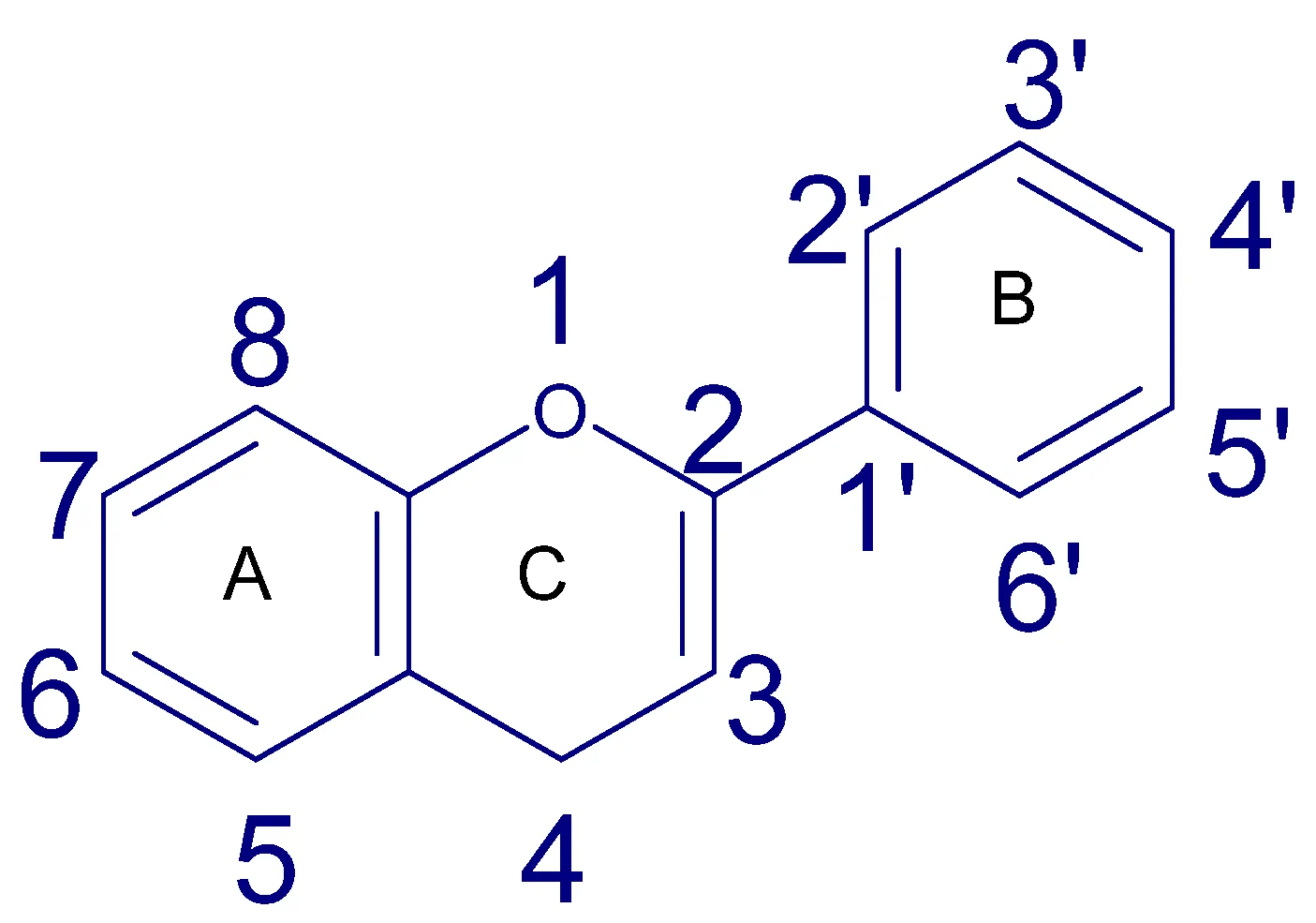
Structural scaffold of flavonoids.

The structural features of flavonoids play a significant role in their diverse biological activities. Structure–activity relationship (SAR) analyses indicate that variations in hydroxylation patterns, conjugation, and glycosylation significantly impact their pharmacological properties. In particular, the presence of a catechol moiety in the B ring and C2-C3 double bond enhances electron-donating ability and radical scavenging activity, which contribute to redox modulation. Glycosylation at the C-3 or C-7 positions (Figure 9), observed in flavonoid glycosides, like rutin, enhances aqueous solubility but may have reduced bioavailability in certain tissues [202,203]. Moreover, hydroxyl substitutions at positions such as C-3 and C-5 promote hydrogen bonding interactions, which may influence binding to regulatory protein targets and protein–ligand complex stability. These structural characteristics affect how flavonoids interact with signaling proteins and their ability to modulate metabolic pathways such as AMPK activation, inflammatory responses, redox signaling pathways, and ACE2-associated pathways relevant to cardiometabolic diseases and viral pathogenesis. Combining computational analyses, SARs, and experimental investigations is pivotal and invaluable in drug discovery. This enables rapid and efficient screening of promising bioactive molecules and the understanding of their potential pharmacological effects.

**Table 1:** Biological compounds with potential AMPK and ACE2 modulation for metabolic and vascular homeostasis.

Compound	Chemical Structure	Metabolic and Inflammatory Targets	Signaling Pathway Modulation	Pharmacological Effects	Potential Indications	Evidence Level	References
Rutin	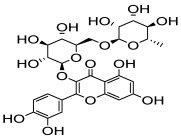	AMPK, PPARγ, MARK, COX-2, NF-κB, TNF-α, IL-6, and IL-1β	Activation of AMPK and PPARγ signaling. Inhibition of TNF-α, IL-6, COX-2, NF-κB, and MAPK pathways	Anti-inflammatory, lipid profile improving, hypoglycemic, antioxidant, and endothelial protective effects	Diabetes, obesity, T2DM, NAFLD, and diabetic complications	Preclinical in vitro and in vivo studies	[204,205]
Quercetin	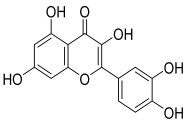	AMPK, NF-κB, and Nrf2 signalling and SREBP pathways	AMPK activation, inhibition of NF-κB, IL-6 ↓, and upregulation of Nrf2	Enhances insulin sensitivity, lipid metabolism, endothelial function, anti-inflammatory effects, and β-cell protection and reduces oxidative stress	Diabetes, T2DM, obesity, insulin resistance, inflammation, and oxidative stress	Limited clinical studies, robust computational, and strong preclinical studies	[183,194,206]
Isoquercetin	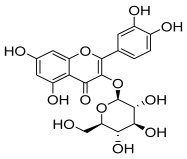	AMPK, PI3K/AKT, NF-κB, and Nrf2 pathways	Activation of AMPK, PI3K/AKT/Nrf2, NF-κB, TNF, and IL-6 inhibition	Diabetes, β-cell protection, inflammation, obesity, and metabolic syndrome	Diabetes, T2DM, inflammation, obesity, and metabolic syndrome	Computational and preclinical studies conducted in vitro and in vivo	[187]
Kaempferol	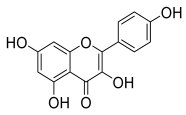	AMPK, PI3K/AKT, NF-κB, and Nrf2 pathways	Activation of AMPK, PI3K/AKT, Nrf2 and inhibition of NF-κB signaling pathways	Decreased oxidative stress, blood glucose, enhanced insulin sensitivity, lipid metabolism, and endothelial function	Diabetes, T2DM, inflammation, obesity, NAFLD, antioxidant, and atherosclerosis	Observational clinical data, computational and preclinical in vitro and in vivo, and underexplored clinical research	[182,196,207]
Isorhamnetin	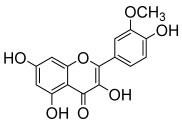	PI3K/AKT signaling pathway and CO-X2	Activation of the PI3K/AKT pathway and inhibition of COX-2	Increased glucose uptake, enhanced β-cell protection, anti-inflammatory effects, and antioxidant activity, and decreased insulin resistance	CKD, T2DM, diabetes and its complications, including inflammation	Computational and preclinical studies in vitro and in vivo, as well as clinical studies that are currently unexplored	[208]
Myricetin	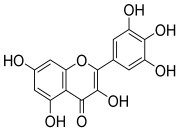	IRS-1/PI3K/Akt/GLUT4, NF-κB, and Nrf2. SARS-CoV-2 Mpro	Activation of IRS-1/PI3K/Akt/GLUT4 and Nrf2. Inhibition of Mpro and viral replication	Increase glucose uptake and insulin sensitivity and reduce blood glucose level, antioxidant, and anti-inflammatory effects	Diabetes, T2DM, and oxidative stress and inflammation	Preclinical studies using in vitro and in vivo models	[184,209]
Apigenin	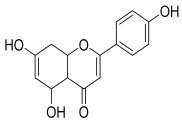	NF-κB, PTP1B, and SREBP. SARS-CoV-2 Mpro	Inhibition of Mpro and viral replication. Prediction of PTP1B inhibition and interaction with the SREBP pathway	Reduced ROS generation, suppressed inflammatory and lipogenic enzymes, improved lipid profile and β-cell protection, and increased insulin sensitivity	Obesity, NAFLD, T2DM, metabolic syndrome, insulin resistance, oxidative stress, and inflammation	Promising preclinical studies, but there is a lack of clinical trials and clinical translation	[175,180,209]
Luteolin	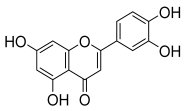	DPP-4, NF-κB	DPP-4 inhibition	Beta-cell protection, reduced oxidative stress, improved aortic and vascular function, enhanced lipid profile and glucose metabolism	T2DM, cardiovascular complications, and oxidative stress	Observational clinical data and preclinical studies have shown promising results	[210]
Baicalein	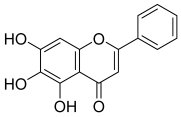	AMPK, SREBP1, NF-κB, SARS-CoV-2 Mpro	Activation of AMPK, inhibition of SREBP1, Mpro, and viral replication	Cytoprotection promotes fatty acid oxidation, antioxidant, anti-inflammatory	Hyperglycemia, NAFLD	Preclinical in vitro and in vivo studies	[211,212]
Resveratrol	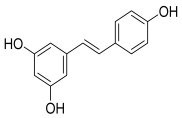	SIRT1, PI3K/AKT/Nrf2 JAK2/STAT3, and NF-Kb,	Activation of AMPK, PI3K/AKT, Nrf2, NF-kB inhibition, IL-6, increase SOD, CAT, GSH, and reduce lipid peroxidation.	Improved insulin sensitivity, glucose metabolism, anti-inflammatory effects, and vascular functions	Diabetes, obesity, insulin resistance, stroke, oxidative stress, and inflammation	Preclinical and small-scale clinical studies	[186,194,213]
Epigallocatechin gallate (EGCG)	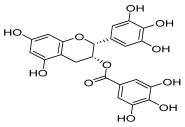	AMPK, NF-κB, and Nrf2	Activation of AMPK and Nrf2 pathways	Decrease oxidative stress and anti-inflammatory effects	T2DM, obesity, NAFLD, and insulin resistance	Preclinical and limited studies	[214]
Metformin	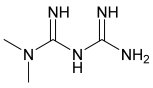	AMPK (indirect), mitochondrial complex I	Indirect AMPK activator; increases cellular AMP/ADP ratio	Reduces endothelial inflammation, improves NO bioavailability, suppresses pro-inflammatory cytokines	Type 2 diabetes, insulin resistance, metabolic syndrome	In clinical use	[201]
Diminazene aceturate (DIZE)	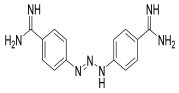	ACE2, AT1R, antagonist, MAPK/ERK, STAT, TNF, and IL-6	ACE2 activator, AT1R antagonist, MAPK/ERK, STAT, TNF, and IL-6 inhibition	Cardiovascular disease, vasodilatation, anti-inflammatory, and atherosclerosis	Endothelial dysfunction, cancer	Preclinical in vitro and in vivo studies	[133]

## 25. Molecular Modeling in Drug Discovery, Repurposing, and the Evolution of Emerging Therapeutics

**Figure 10:**
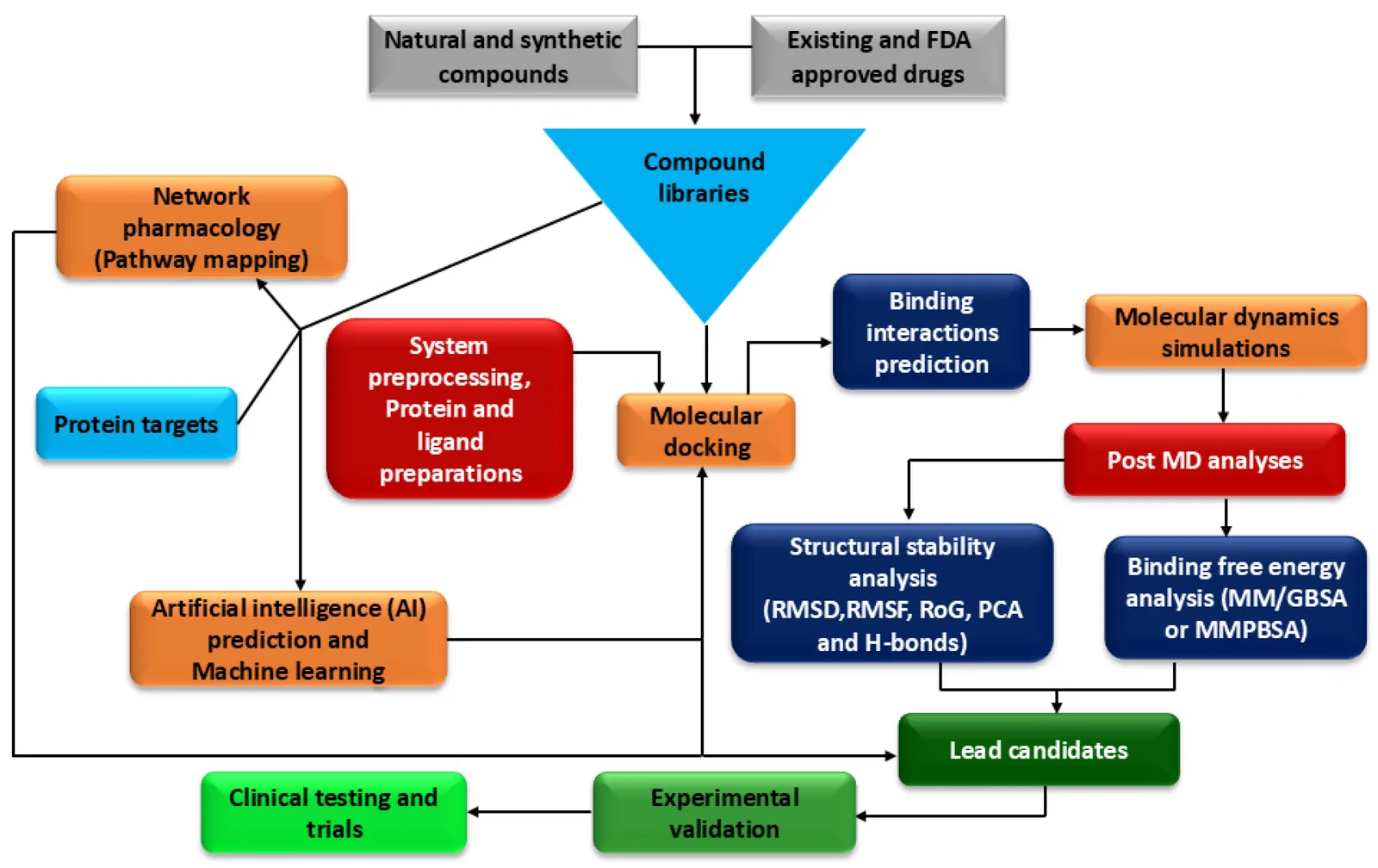
Computational methods for identifying targets and lead candidates, leading to preclinical and clinical testing.

Molecular modeling rapidly emerged as a cornerstone in drug repurposing and the development of novel therapeutics, particularly in response to global health crises, such as the COVID-19 pandemic. These computational techniques, ranging from virtual screening and molecular docking to molecular dynamics (MD) simulations and binding free energy calculations, allow rapid, cost-effective, and mechanistically insightful identification of drug candidates. These techniques facilitate the systematic exploration of interactions between small molecules and biological targets. This helps to gain insight into binding affinity, intermolecular interactions, structural stability (Figure 10), and potential pharmacological activity. Advanced systems-level methods such as network pharmacology analyses, machine learning, and artificial intelligence (AI) prediction models further support multi-target analysis and virtual screening for candidate identification [18,215]. These methods are extensively used to study natural bioactive compounds, approved drugs, and synthetic compounds targeting proteins involved in metabolic diseases, viral pathophysiology, and emerging diseases. The integration of these computational methods facilitates rapid identification and prioritization of potential therapeutic agents for existing and emerging diseases.

Several studies demonstrated the power of molecular modeling in repurposing drugs against SARS-CoV-2 early in the pandemic. Drug repurposing accelerates the development of new treatments by utilizing the safety profiles of existing drugs. Baby [17] employed a dual-targeting strategy to screen FDA-approved drugs for entry inhibitors against SARS-CoV-2 using the Schrodinger suite, identifying lopinavir and valrubicin as having superior binding affinities and dual inhibition for both TMPRSS2 and ACE2. Targeting both proteins showed significantly promising results for preventing viral entry [17]. This study highlights the significance of a dual-targeting approach for effective therapy and computational investigations in identifying leads or repurposing existing drugs for further preclinical evaluations or clinical applications. Buitrón-González [216] explored the repurposing potentials of amprenavir, enalaprilat, and plerixafor as potential destabilizers of the SARS-CoV-2 spike protein–ACE2 complex using molecular docking and MD simulations [216]. Their study reported that plerixafor formed the most stable interactions with the S protein–ACE2 interface, suggesting strong potential for repurposing and therapeutic applications. Kumar Verma [217] investigated Ayurvedic medicinal plant-derived compounds using molecular docking, MD simulations, and MM/PBSA and highlighted the integration of traditional medicine with computational tools [217]. Further validation using a Bayesian machine learning model using ECFP6 fingerprints showed that gingerol, quercetin, and curcumin are promising against ACE2 and SARS-CoV-2 target proteins [217]. Patel [218] showed that phytochemicals could hinder the interaction of the SARS-CoV-2 S protein with ACE2, adding evidence for natural product-based repurposing [218].

Another key contribution and focus was on viral enzymes. Pitsillou [219] used molecular modeling to screen 300 compounds against the nidovirus RdRp-associated nucleotidyltransferase (NiRAN) domain of SARS-CoV-2 RNA-dependent RNA polymerase (RdRp), identifying small molecule inhibitors with strong binding energies, including oleuropein as a lead compound and potential allosteric sites at the nsp12–nsp8 interface region [219], while Mahdian [220] repurposed FDA-approved drugs using combined molecular docking and MD simulations, identifying vancomycin, ledipasvir and estradiol benzoate as promising inhibitors against RdRp and host receptors ACE2 and CD147, respectively [220]. Expanding therapeutic targets, Zhang [221] identified drugs targeting cell surface binding immunoglobin protein (csBiP) and reported that targeting csBiP offers a novel therapeutic axis to disrupt SARS-CoV-2 binding and replication [221]. Studies increasingly incorporated advanced techniques. Yang [222] employed a deep learning-based machine learning model that utilizes Chemprop MPNNs and in vitro assay screening to target Cathepsin L (CTSL) with FDA-approved drugs. Their study identified five promising molecules with predicted nanomolar inhibitory affinities against CTSL [222]. Lazniewski [223] performed extensive MD simulations, showing that several FDA-approved drugs disrupted spike–ACE2 interactions with RMSD deviations exceeding 2.5 Å [223]. This study indicated the growing importance of long-scale dynamics.

Employing deeper computational analyses, in silico pipelines became more comprehensive. MacMahon [215] developed an in silico pipeline to identify drugs that are structurally similar to the COVID-19 clinical trial drugs (C19-CTDs) that might inhibit SARS-CoV-2 replication by integrating structure-based screening with network biology and pathway enrichment analyses. The study screened over 7682 compounds and identified 101 compounds that are structurally similar to C19-CTDs, identifying triamcinolone and gallopamil as promising candidates that could potentially modulate early viral replication by targeting kinase activity [215]. Lou [224] introduced knowledge graph embedding models for identifying potential targets for drug repurposing. Through a knowledge integration approach, the study identified 33 potential targets and 18 drug candidates with potential activity against coronavirus. The study indicates the effectiveness of using biomedical knowledge networks and computational methods in uncovering the pathogenicity and transmission mechanisms of emerging viruses [224]. Ahmed [225] used structure-based in silico screening and covalent docking to identify FDA-approved drugs for targeting SARS-CoV-2. In vitro antiviral evaluation of the top candidates identified mebendazole and atovaquone as promising candidates based on their IC_50_ values and pharmacokinetic properties. Mebendazole showed significant inhibition against Mpro activity, while atovaquone showed significant inhibition against viral replication. The study emphasized the potential of repurposing FDA-approved drugs for treating diseases through structure-based approaches and host-specific mechanisms [225]. Experimental validation began to complement computational insights. Khan [226] integrated molecular docking, MD simulations, STD-NMR spectroscopy, and antiviral assays to repurpose 156 compounds against SARS-CoV-2 Mpro. They evaluated the stability, interactions, and thermal modulation of Mpro as well as the potential antiviral inhibitory activity of the compounds. The study identified 10 FDA-approved drugs that interacted with the viral protein and showed therapeutic potential in inhibiting SARS-CoV-2 [226]. Lin [209] evaluated the antiviral potential of flavonoids, showing the inhibition of Mpro by demonstrating that apigenin and pinocembrin 7-O-(3″-galloyl-4″,6″-(S)-hexahydroxydiphenoyl) β-D-glucose (PGHG) potently suppressed SARS-CoV-2 replication in vitro using a Vero E6-based SARS-CoV-2 replication assay and in silico methods [209]. This integration of in silico and experimental methods marked a critical evolution towards transitional applicability.

Computational investigations have increasingly shifted towards network-based and higher-resolution imaging analyses. In a recent study by Bogacheva [227], high-throughput (HT) cell viability, imaging, and in silico analysis were utilized to identify potential therapeutic compounds against SARS-CoV-2. The study, through in silico analyses using the Galaxy Chemical Annotation Retrieval Toolkit (CART), identified 11 cellular proteins as significantly enriched drug targets for 19 promising molecules. By mapping the host–virus specific protein interaction mechanism, the in silico interaction analysis identified duvelisib as a potential drug candidate that targets the SARS-CoV-2 interactome without causing phospholipidosis [227]. This high-throughput network-based approach of computational and in vitro analysis facilitates rapid screening and repurposing of existing drugs for the accelerated discovery of effective therapies against emerging diseases, particularly viral infections like COVID-19. Avilés-Alía [228] used an ensemble docking process and binding free energy calculations to identify the top 10 most promising FDA-approved drugs as potential S protein inhibitors. The study found that estradiol cypionate and telmisartan effectively destabilized the spike–ACE2 binding interface and reduced viral replication in ex vivo testing [228]. In the study by El Sobky [229], computational analysis was used to screen FDA-approved drugs that could inhibit ACE2-spike protein binding for in vitro antiviral assessment. The study identified three clinically relevant drugs, argatroban, glimepiride, and ranolazine, that have shown anti-SARS-CoV-2 activity through diverse mechanisms, including Mpro inhibition and host factor modulation [229]. This study illustrated the potential of molecular modeling in identifying promising therapeutic options from the existing therapeutics. 

The study by Bakshi [18] demonstrates the effectiveness of multiscale integration in drug repurposing for combating complex pathophysiological conditions, like the COVID-19 cytokine storm. The study utilized state-space modeling, parameter estimation, deep learning models, and molecular docking to quantitatively model cytokine network dynamics and identify controllable system parameters linked to drug–target interactions. The approach demonstrates the effectiveness of a shift from single targeting toward a system-level targeting of critical regulatory proteins in disease pathways, identifying ACE2 activation and AT1R inhibition as promising therapeutic strategies in mitigating hyperinflammation caused by SARS-CoV-2. The study presents an approach with significant potential for developing targeted therapies, predicting drug efficacy, safety, and understanding biological interactions across different diseases [18]. The study by Chen [230] used computational methods and in vitro assays to show that dibutyl phthalate inhibits various SARS-CoV-2 variants through allosteric disruption of the spike–ACE2 interface, independent of orthosteric competition. This suggests the potential of the allosteric site as a robust repurposing strategy against viral mutations [230]. Complementarily, Pattis [231] further explored this concept by employing molecular dynamics simulations with flooding fragments (MDFFr) to identify allosteric and cryptic pockets in the SARS-CoV-2 papain-like protease (PLpro), providing important insights into overcoming resistance mechanisms [231]. The evolution of centric approaches in drug repurposing is extensively discussed by Cong [232] and Wang [233]. They outline artificial intelligence (AI) and machine learning (ML)-driven drug repurposing pipelines that involve data curation, feature learning, target prediction, and lead optimization. These studies establish AI as a unifying layer that integrates QSAR, docking, and systems modeling to drive therapeutic innovation [232,233].

Drug repurposing is a complex and multifaceted process that involves molecular modeling techniques such as allostery, molecular docking, dynamics simulations, QSAR, AI, and experimental validation to develop innovative therapeutics based on a solid understanding of the underlying mechanisms and existing safety data.

## 26. Conclusions

Growing evidence identifies the AMPK-ACE2 axis as a pivotal signaling node linking metabolic regulation, vascular health, immune modulation, and viral pathophysiology. This study examines findings demonstrating the interconnected roles of AMPK and ACE2 across metabolic, cardiovascular, inflammatory, and viral pathogenesis. AMPK phosphorylation enhances ACE2 stability and activity, while ACE2 signaling reciprocally promotes AMPK activation, establishing a protective loop that maintains metabolic and vascular homeostasis. Disruption of this regulatory axis has shown a mechanistic explanation for the close association between metabolic dysfunction and increased cardiovascular and viral vulnerability, particularly in diabetes, obesity, and metabolic syndrome. Impaired AMPK-ACE2 signaling promotes insulin resistance, oxidative stress, endothelial dysfunction, and chronic inflammation, which are central to the development and progression of cardiometabolic diseases and heighten susceptibility to severe viral infections, such as COVID-19. Noticeably, SARS-CoV-2 degradation of ACE2 through spike protein binding profoundly perturbs AMPK-ACE2 signaling, leading to impaired AMPK activity, vascular injury, increased inflammation, and metabolic deterioration, exacerbating disease severity, particularly in individuals with pre-existing metabolic disorders. The COVID-19 pandemic has shown how metabolic dysfunction impacts infectious disease outcomes.

The AMPK-ACE2 axis, as a molecular interface that bridges metabolic diseases and viral infection, is a significant therapeutic target. Hence, pharmacological activation of AMPK-ACE2 signaling offers a substantial and promising therapeutic strategy to counteract these interconnected pathological processes. AMPK activation by bioactive molecules like metformin with pleiotropic effects, including metabolic, vascular, and anti-inflammatory benefits beyond the glucose-lowering effects, offers immediate opportunities for drug repurposing and translation while exploring novel AMPK activators and ACE2 modulators. Similarly, bioactive molecules, such as flavonoids with multifunctional therapeutic properties and targets, are also promising natural candidates for dual modulation of AMPK activation and ACE2 expression or stability. However, experimental evidence supporting flavonoid effects on ACE2 phosphorylation and stability is still limited, necessitating further investigations.

The convergence of metabolic and infectious disease pathophysiology through AMPK-ACE2 highlights the significance of comprehensive approaches to understanding and managing intricate diseases. Focusing on this pathway offers a scientifically sound and clinically significant strategy that could enhance outcomes for the increasing prevalence and impact of metabolic and infectious diseases on vulnerable populations. Hence, future investigations should advance AMPK-ACE2 signaling and prioritize integrating mechanistic preclinical and clinical studies to establish therapeutic applicability, pharmacokinetic behaviour, and safety profiles of dual AMPK-ACE2 modulators with biomarkers of pathway activation. Targeting the AMPK-ACE2 axis holds strong promise as a rational and innovative framework for the development of broadly applicable therapies capable of mitigating the complex interplay between metabolic diseases and viral disease severity. In addition, flavonoids represent a group of natural compounds capable of targeting the intertwined pathophysiology of cardiometabolic diseases and severe viral infections, such as COVID-19, by activating AMPK signaling and potentially modulating ACE2-associated mechanisms.

## Data Availability

No new data were created or analyzed in this study. Data sharing is not applicable to this article.
